# Lessons learned from immunological characterization of nanomaterials at the Nanotechnology Characterization Laboratory

**DOI:** 10.3389/fimmu.2022.984252

**Published:** 2022-10-10

**Authors:** Marina A. Dobrovolskaia

**Affiliations:** Nanotechnology Characterization Laboratory, Cancer Research Technology Program, Frederick National Laboratory for Cancer Research, Sponsored by the National Cancer Institute, Frederick, MD, United States

**Keywords:** nanoparticles, nucleic acids, immunotoxicity, characterization, trends, methods

## Abstract

Nanotechnology carriers have become common in pharmaceutical products because of their benefits to drug delivery, including reduced toxicities and improved efficacy of active pharmaceutical ingredients due to targeted delivery, prolonged circulation time, and controlled payload release. While available examples of reduced drug toxicity through formulation using a nanocarrier are encouraging, current data also demonstrate that nanoparticles may change a drug’s biodistribution and alter its toxicity profile. Moreover, individual components of nanoparticles and excipients commonly used in formulations are often not immunologically inert and contribute to the overall immune responses to nanotechnology-formulated products. Said immune responses may be beneficial or adverse depending on the indication, dose, dose regimen, and route of administration. Therefore, comprehensive toxicology studies are of paramount importance even when previously known drugs, components, and excipients are used in nanoformulations. Recent data also suggest that, despite decades of research directed at hiding nanocarriers from the immune recognition, the immune system’s inherent property of clearing particulate materials can be leveraged to improve the therapeutic efficacy of drugs formulated using nanoparticles. Herein, I review current knowledge about nanoparticles’ interaction with the immune system and how these interactions contribute to nanotechnology-formulated drug products’ safety and efficacy through the lens of over a decade of nanoparticle characterization at the Nanotechnology Characterization Laboratory.

## Introduction

**Figure 1:**
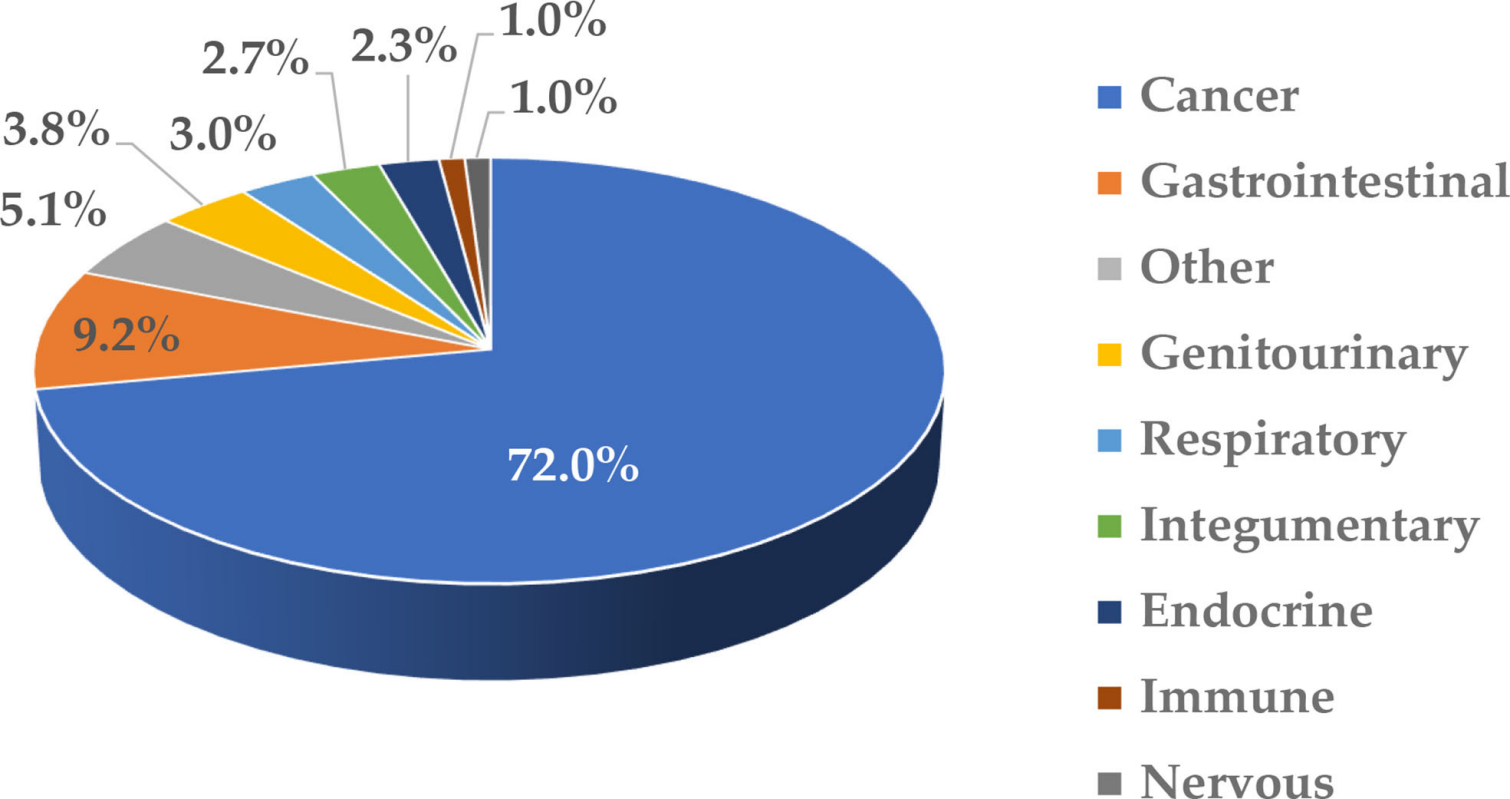
Clinical trials involving nanotechnology-based formulations. This figure was prepared based on data downloaded from ClinicalTrials.gov (8) on August 5, 2020; the accessibility of the site was verified on March 2, 2022. The data were grouped based on disease type and the percentage of total (1,200) was calculated. The cancer category included malignancies affecting various organs and systems. Non-cancerous diseases were grouped based on the type of affected system. “Other” includes communicable diseases, congenital abnormalities, body weight, musculoskeletal, death, fibrosis, infectious and stomatognathic diseases, tissue adhesion, blister, and breast and otorhinolaryngologic diseases.

Nanotechnology is often used to formulate various drugs to improve their solubility, prolong circulation time, achieve delivery to the target organs and tissues, direct the route of particle uptake into and intracellular distribution within a target cell, and benefit from multifunctional capabilities (1–6). Many nanotechnology-based concepts are already used in the clinic and include, among others, anticancer formulations (e.g., Onivyde, Doxil, Abraxane, Daunoxome), anti-microbial agents (e.g., Ambisome), therapeutic nucleic acids (e.g., Onpattro), and vaccines (e.g., Comirnaty). Some industry reports suggest that the global nanomedicine market is rapidly increasing at a compound annual growth rate of 12.6% and will reach $258.11 billion in 2025 (7). Indeed, many nanotechnology-based concepts are in various stages of drug development, including clinical trials. As a recent example, in August 2020, ClinicalTrials.gov reported 1,200 various nanoparticle-based treatments for over 200 indications (8); these numbers continue to grow every year. Most of these concepts (~72%) in 2020 were intended to treat different cancer types, while a small percentage covered indications for body weight, non-cancerous diseases affecting various systems, and infectious diseases (**Figure 1**). The dominance of anti-cancer nanomedicines is not surprising due to the extensive research in the past three decades demonstrating the role of the enhanced permeability and retention (EPR) phenomenon in nanoparticle trafficking to and accumulation in solid tumors. The initial EPR concept implied that due to their size, nanoparticles readily pass the leaky vasculature of tumors and stay in the tumor milieu, unable to exit quickly due to altered lymphatic drainage; as such, they accumulate and release drugs in tumors, reducing the exposure of healthy tissues to cytotoxic drugs. However, more recently, the complexity of EPR became evident in that this phenomenon was more pronounced in some but not all solid tumors and varied considerably between patients. This recent notion stimulated cancer nanomedicine researchers to develop strategies, such as quantifying the degree of EPR in individual patients by non-invasive imaging techniques prior to administering the treatment, with the overall goal of improving the delivery of nanomedicines to tumors. The controversy surrounding EPR and various strategies for improving cancer nanomedicine targeting and efficacy have been recently discussed by Lammers and the team (9). Verifying the clinical utility of these strategies is expected to result in more cancer nanomedicine concepts going into clinical trials.

Given the current global emergency use of lipid-nanoparticle (LNP)-based COVID-19 vaccines to combat the COVID-19 pandemic, some experts expect more nanotechnology applications in infectious diseases (10).

Many studies have demonstrated that the reformulation of a drug using a nanocarrier helps to reduce the drug’s toxicity. For example, the anticancer drug doxorubicin (DXR) is known for its accumulation in cardiomyocytes and in relation to its cardiotoxicity; this toxicity is overcome when DXR is delivered using a polyethylene glycol (PEG)-modified liposome (Doxil) (11). The removal of toxic excipient Cremophor and reformulation of another anticancer drug, paclitaxel, using nanoalbumin particles, resulted in an improved safety profile. As a result, the original, Cremophor-EL-based formulation of paclitaxel (Taxol) requires slow infusion and premedication to avoid anaphylactoid reactions, whereas nanoalbumin-formulated paclitaxel (Abraxane) is injected without premedication and does not induce anaphylaxis (12, 13). The formulation of therapeutic proteins TNFα and coagulation factor VIII (FVIII) using colloidal gold and liposome, respectively, helped to overcome systemic inflammatory response to TNFα and generation of neutralizing antibodies to FVIII (14–16).

While reduced drug toxicity through formulation with a nanocarrier is encouraging, available data also suggest that a change in the drug’s biodistribution due to the carrier may also occur and lead to the “relocation” of toxicity from one target organ to another. For example, the liposomal formulation of DXR helped to overcome DXR’s cardiotoxicity (11); however, due to the liposomal drug’s accumulation in the skin’s dendritic cells, it created new toxicity, palmar-plantar erythrodysesthesia (PPE, also known as Hand-and-Foot Syndrome) (17). Reformulation of the same active pharmaceutical ingredient (API) DXR using cyanoacrylate nanoparticles eliminated cardiotoxicity and PPE but resulted in nephrotoxicity due to the drug’s accumulation in the kidneys (18). These studies emphasize the importance of performing comprehensive toxicology studies even when a previously known drug is used in formulations using nanoparticles because nanocarriers may change the distribution of the drug and, hence, alter its toxicity profile.

For decades, the efforts of the nanotechnology drug delivery community were focused on masking nanoparticles from immune recognition (19–21). However, recent evidence shows that this intrinsic ability of the immune system to clear particulate materials—one that researchers have tried to work around for years—is one that can be modulated to synergize with the primary mechanism of action of drugs delivered by nanocarriers. This creates limitless opportunities to harness this property and direct it against disease-causing mechanisms (22). For example, PEGylated liposomal DXR (Doxil), initially approved for cancer therapy due to its ability to decrease DXR-mediated cardiotoxicity, is now known to stimulate the anticancer immune response, through a mechanism that is not completely understood, that allows for improved anticancer efficacy when Doxil is combined with immune-checkpoint inhibitors (23). A study in a colorectal cancer model in immunocompetent but not immunocompromised mice demonstrated that a combination of Doxil and anti-PD1 resulted in a complete response in 11 out of 12 animals (23). In another study, the same API DXR, formulated using polymeric-LNPs, was effective against breast cancer in treated animals by reducing immunosuppression in the tumor microenvironment (24). RGD-targeted LNPs co-delivering API (a-GalCer) and an immunomodulatory agent (PI3K inhibitor) improved therapeutic outcomes against breast tumors (25). Nanoalbumin-formulated paclitaxel (Abraxane) that has already been approved for clinical use as monotherapy is also undergoing clinical testing in combination with immune-checkpoint inhibitors to improve the outcome of anti-tumor therapy (26, 27).

The mechanisms through which nanocarriers contribute to immunomodulation are incompletely understood. One mechanism commonly discussed in cancer therapy literature includes the induction of so-called immunogenic cell death (ICD) by APIs, which are more precisely delivered to tumors by nanocarriers (28). The studies that favor this mechanism include those demonstrating that APIs, such as paclitaxel, oxaliplatin, gemcitabine, DXR, 5-fluorouracil, and gemcitabine, to name a few, activate apoptotic pathways that lead to the release of so-called danger signals or danger-associated molecular patterns [DAMPs (e.g., ATP, calreticulin and high-mobility group-B1 protein)] that activate tumor-infiltrating antigen-presenting cells, thereby contributing to immunogenicity of tumor-specific antigens released by dying cancer cells; these studies have been discussed in detail elsewhere (29–31). In contrast, some studies clearly demonstrate that cytotoxic APIs do not have the same efficacy as their nanoparticle-formulated counterparts when used in immunocompetent animals and combined with immune-checkpoint inhibitors (23). Therefore, ICD induction by cytotoxic APIs alone does not entirely explain the observed improvement in anti-tumor efficacy.

The existing data suggest that nanocarriers may contribute to this phenomenon through other mechanisms than delivering an ICD-inducing drug to tumors. Some of such mechanisms may include nanocarriers inducing chemokines. For example, I reported earlier that liposomes and lipid nanocarriers commonly induce chemokine IL-8 (32), which is responsible for the recruitment of leukocytes (33). Other mechanisms may be linked to intracellular complement activation. For example, our team found that dendrimers and other cationic polymeric molecules activate an intracellular complement (34) that plays a critical role in regulating T-cell activation (35–37).

Moreover, nanocarriers can be loaded with immunomodulatory agents that improve the therapeutic outcome of cytotoxic agents. For example, liposomes formulated to co-deliver a PI3K inhibitor with an API (a-GalCer) activated anti-tumor T-cell responses (25). In another recent study, nanoscale coordination polymer core-shell nanoparticles were designed to co-deliver oxaliplatin and dihydroartemesinin; these particles induced reactive oxygen species (ROS), which activated the immune cells and improved the anti-tumor response to anti-PD-1 immunotherapy (38). Interestingly, chemokine induction by lipid-based nanocarriers has also been attributed to their ability to induce ROS (39). Besides activating the chemokine responses, oxidative stress also negatively regulates the complement factor H (a complement system inhibitor), thereby further contributing to inflammatory responses (40). Therefore, oxidative stress induced by a nanocarrier may be an important mechanism contributing to the observed efficacy of nanoformulated drugs in immunotherapy applications.

It is well established now that nanoparticle physicochemical properties such as size, aspect ratio, zeta potential, hydrophobicity, surface area, and functionalization determine interactions between nanoparticles and immune cells. By optimizing these properties, researchers could control undesirable immunotoxicity and achieve desirable immunomodulatory effects. More studies are needed to fully understand the mechanisms by which nanocarriers contribute to API therapeutic efficacy (besides their primary role as drug-delivery vehicles).

Regardless of the indication, all new formulations must undergo rigorous safety testing prior to their approval for clinical use. Even after receiving initial approval, drugs undergo post-marketing surveillance and can be removed from the market due to toxicity (41). One reason for drug discontinuation in clinical practice is immunotoxicity, with hypersensitivity reactions (HSRs) being named frequently (42, 43). Herein, I will focus on available information relevant to HSRs and immunosuppression and review the current literature about nanoparticle-mediated immunotoxicity and available methodologies to study it.

## Infusion reactions

**Figure 2:**
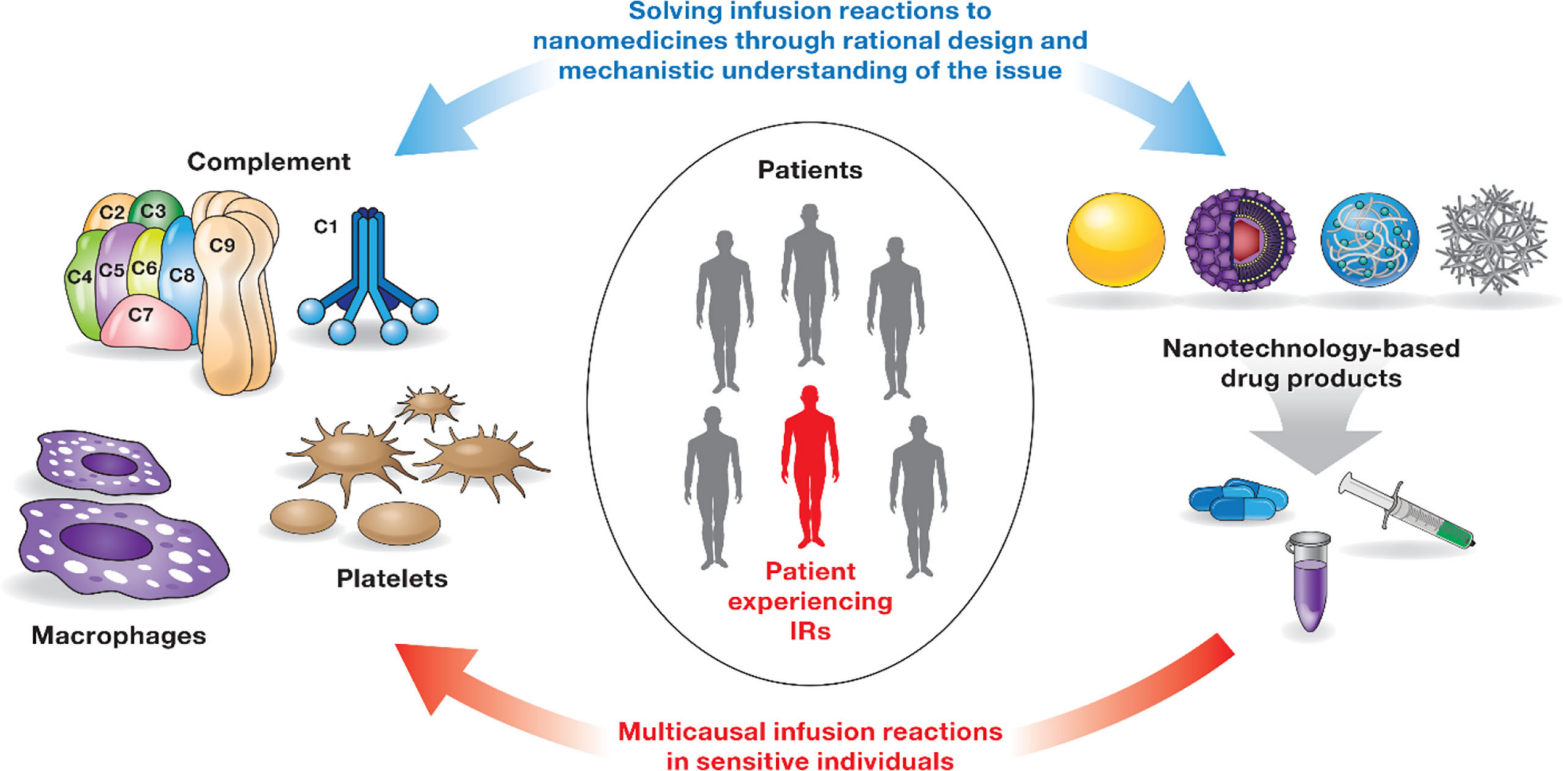
Infusion reactions to nanomedicines. IRs to nanomedicines involve multiple players (immune cells, coagulation, and complement systems) and complex, often overlapping mechanisms. Not all patients are sensitive to these responses. Interaction between one or several components of the immune system and the nanocarrier triggers these responses in sensitive individuals. Timely detection and appropriate management of IRs are critical to avoid severe health consequences for patients undergoing therapy with nanomedicines. IRs are not unique to nanomedicines and have been documented for other types of drug products (44). Rational design of nanocarriers and understanding of mechanisms underlying nanoparticle-mediated immunotoxicity are currently considered a solution to overcome the issue of IRs to nanomedicines.

Infusion reactions (IRs) are HSRs that occur within minutes to hours of nanoparticle administration (44). The mechanisms underlying IRs to nanomedicines are complex and often involve overlapping pathways and systems (**Figure 2**). Some of the currently known mechanisms include activation of the complement system and so-called complement activation related pseudoallergy (CARPA), activation of platelets that release secondary mediators contributing to the overall response, and production of cytokines by immune cells, including but not limited to macrophages (44–48). Interestingly, IR symptoms in patients receiving intravenous (i.v.) injection or infusion of nanomedicines overlap with that of HSR in individuals immunized with LNP-based mRNA vaccines (49). It is generally agreed that the same pro-inflammatory properties of LNPs required for vaccine efficacy also contribute to the HSR. More detailed mechanisms and safety roadmaps for IRs to nanomedicine and HSRs to LNP-based mRNA vaccines have been discussed elsewhere (44, 49). Below I will focus on the complement system, the coagulation system, and cytokines that are recognized among leading contributors to nanoparticle-mediated IRs and HSRs.

## Complement system

**Figure 3:**
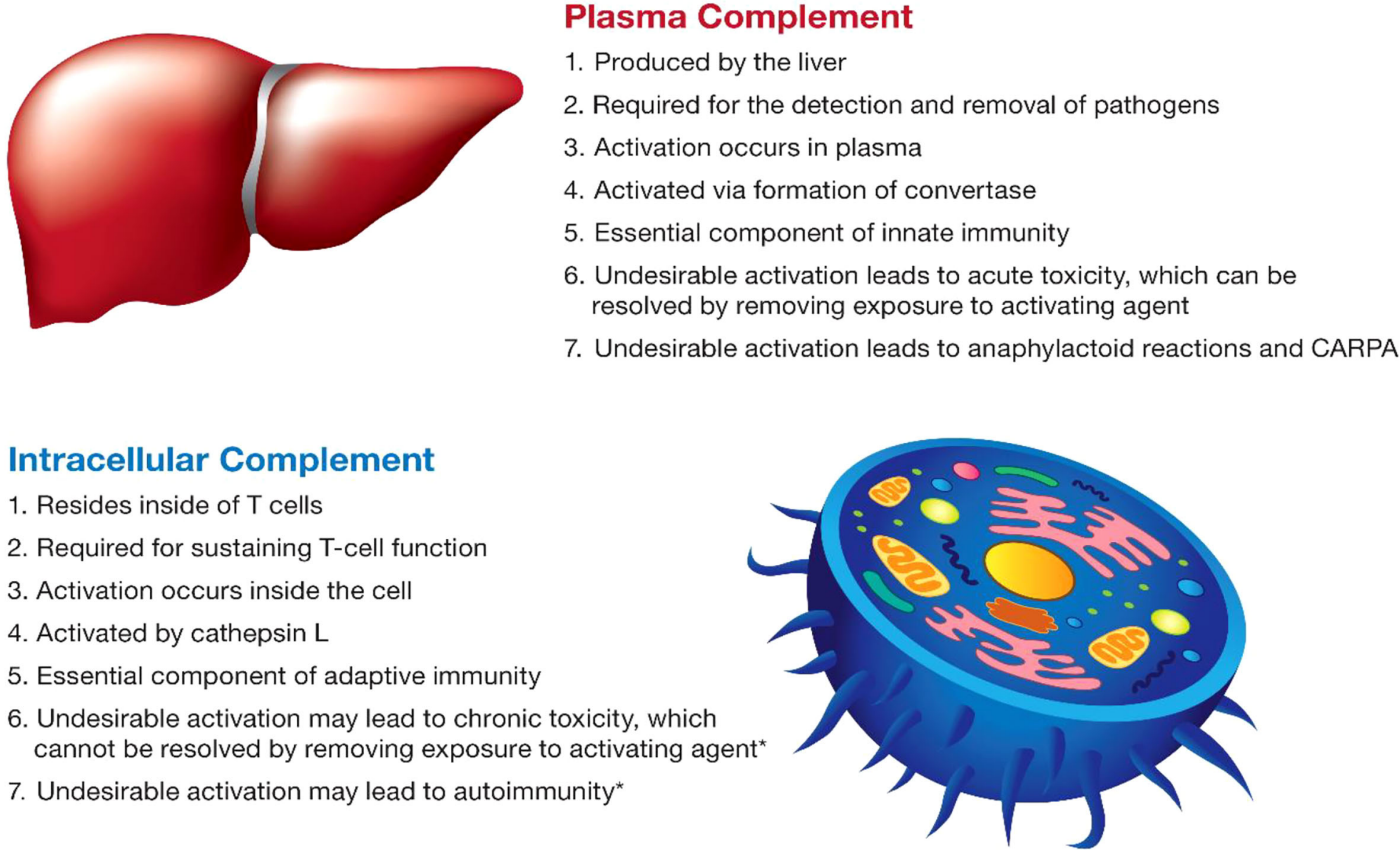
The main characteristics of the complement system. The main characteristics of plasma and intracellular complement are summarized. The intracellular complement system is discussed in the figure in the context of T lymphocytes due to a better understanding of its function in the currently available literature. The intracellular complement system has also been detected in other cell types; its role in other cells is less understood and, therefore, not mentioned in the figure. Statements highlighted with an asterisk (*) were hypothesized based on the role of lymphocytes in which the intracellular complement system was described; experimental verification is still required for these statements and represents one of the future directions of research in this field.

The complement system plays an essential role in both innate and adaptive immunity (50). It is complex and includes a large group of proteins that are produced by different cells in the body, act in different compartments, get activated by different mechanisms, and contribute to different types of immune responses (**Figure 3**). The discussion below focuses on current knowledge about plasma and intracellular complement systems.

### Plasma complement

The plasma complement is a group of more than 30 proteins produced by the liver and secreted into the blood, where they “complement” cellular immune defense mechanisms. Activation of the plasma complement system occurs *via* three main mechanisms—the lectin pathway, initiated by mannose-binding lectin; the classical pathway, initiated by the antibody; and the alternative pathway initiated by C3b binding. Additionally, autoactivation can occur *via* the so-called C3-tickover mechanism (51). Once triggered, these pathways result in sequential proteolytic cleavage of complement proteins organized in a cascade that converges on the C3 component of the complement. The activation culminates with the formation of terminal, or so-called membrane-attack complex, sC5b-9, which is perforin disrupting a microbe membrane and “killing” the microbe. Activation of the plasma complement results in production of anaphylatoxins—C3a, C4a, and C5a—that act like cytokines and activate immune cells, thereby promoting the immune response (51). The action of the plasma complement system is tightly connected to that of the blood coagulation and kinin/kallikrein systems, collectively acting to stop the infection and restore homeostasis. The same components of the complement system intended for the elimination of the pathogen—anaphylatoxins and terminal complex—are also responsible for adverse effects: tissue swelling, redness, pain, and cardiopulmonary changes. When complement activation is triggered by drug products (e.g., PEGylated liposomal DXR or Cremophor-EL formulated drugs), it leads to CARPA. CARPA symptoms overlap with that of immediate type I HSRs triggered by antigen-specific IgE. When left uncontrolled, CARPA may be fatal. Janos Szebeni of the Semmelweis University in Hungary pioneered the research on CARPA; he coined the term, described the mechanism, and developed *in vitro* and *in vivo* models used by other researchers worldwide to understand this phenomenon further and find the means for controlling it to prevent adverse health effects. Plasma complement activation and CARPA in response to pharmaceuticals, including those formulated using nanotechnology, have been extensively discussed in the literature (most recent references: (44, 46, 48, 52–54); Dr. Szebeni published more than 100 papers on this subject). Understanding the immunogenicity of drug products and their components, as detailed in the immunogenicity section of this review, provides mechanistic insights in understanding the CARPA phenomenon due to the known role of certain types of antibodies in activating the classical complement pathway.

Herein, I want to briefly summarize key structure-activity relationships and current approaches for minimizing the ill effects of CARPA on patients receiving nanomedicines to lay the foundation for the next section pertaining to the lesser-known intracellular complement system. Factors influencing complement activation by PEGylated liposomes include lipid composition and structure, zeta potential, surface and PEG phospholipid anchor charge, density, and the molecular weight of PEG (55). Similar findings were described in another study demonstrating that conformation and density of glycopolymer coating on polystyrene nanoparticles can serve as “molecular switches” of complement activation (56). An increase in the surface load of cationic moieties on perfluorocarbon nanoparticles was associated with an increase in complement activation, whereas the addition of PEG-3000, but not PEG-350, decreased the reactogenicity (57). Moreover, drug release and crystal formation at the particle surface and contamination with endotoxin may further contribute to the reactogenicity of nanoparticles with the complement system, as was discussed in a liposome study (55). Decreasing nanomedicine infusion rate *in vivo*, applying complement inhibitors, and injecting empty nanocarriers (e.g., Doxebo) before administering drug-loaded nanoparticles (e.g., Doxil) were proposed as effective means of inhibiting complement activation by nanoparticles *in vitro* and *in vivo* (58–60).

Among nanoparticles that passed characterization in the Nanotechnology Characterization Laboratory (NCL; https://ncl.cancer.gov/) assay cascade, PEGylated liposomes, especially those with elongated shapes, had more significant complement activation responses than spherical PEGylated liposomes and other PEGylated nanomaterials, which is consistent with the literature (55). The significant factors determining the nanoparticles’ complement activating ability in the NCL assay cascade include composition, shape, and dose. While we found that anti-PEG antibodies contribute to complement activation by PEGylated liposomal DXR, we observed no correlation between the anti-PEG antibody titer in the normal donors’ blood and the magnitude of the complement activation (61). We concluded that the presence of antibodies might be monitored for mechanistic purposes when the reaction occurs, but it should not be used to predict the reaction; instead, functional assays such as an *in vitro* complement activation assay are a more accurate tool to identify nanoparticles that trigger complement activation *in vitro*, and, as such, have a greater risk of causing CARPA *in vivo*.

### Intracellular complement

Unlike the plasma complement proteins produced in the liver and secreted into the blood, the intracellular complement system is expressed by and remains inside the cells (**Figure 3**). The expression is either constitutive or induced by stimuli that activate the cells (35–37, 62). Once activated, intracellular complement split products are transported outside the cell and are exposed on the cellular membrane (34–37). Both C3 and C5 components of the complement system were described in cells. Even though the intracellular complement system is more extensively studied in T cells, it is not specific to T-lymphocytes and is found in other cell types including immune cells (monocytes, neutrophils, and B cells), non-immune cells (epithelial cells, endothelial cells, fibroblasts, and adipocytes), and cells that have undergone malignant transformation (62–64). The intracellular C3 component of the complement system produced by dendritic cells contributes to T-cell activation (65); when expressed by cancer cells, it promotes tumor growth *via* a mechanism involving the PI3K/Akt pathway (64). The autocrine activation of CD46 and C3aR by intracellular complement directs the metabolic reprogramming of T-cells and determines the Th1 polarization phenotype of activated T-lymphocytes (63, 66).

The study by Liszewski et al. identified the protease cathepsin L as the enzyme responsible for cleaving the C3 protein and generating the C3a split product to be exposed on the cellular membrane (63). Another study was unable to reproduce this mechanism despite analyzing the same activating stimulus (a-CD3 antibody) (34), suggesting that multiple mechanisms of intracellular complement activation likely exist.

Nanoparticles activate intracellular complement based on their surface charge. A recent study investigated a large group of nanomaterials for their ability to trigger intracellular complement activation in human cells (34). The study organized test nanomaterials into several groups based on current knowledge of their involvement in different types of immunotoxicity. One group included materials known for their ability to activate the plasma complement system and cause CARPA in sensitive individuals (e.g., PEGylated liposome, amphotericin-loaded liposome Ambisome, and iron oxide nanoparticles (IONPs) Feraheme, polyethoxylated castor oil Cremaphor-EL, and Propofol). Another group included nanomaterials with an established record of delayed-type HSR and contribution to protein immunogenicity (e.g., nickel, zinc oxide, gold, and silver nanoparticles). The third group was based on materials with a known record of perturbation or disruption of cellular organelles (silica, silicon, nano-silica particles, and dendrimers). Among these materials, only amine- and guanidine-terminated polyamidoamine (PAMAM) and amine-terminated triazine dendrimers activated the intracellular complement system in manner dependent on size and density of surface groups (34). In all cases, complement split products C3c and C3d were detected on the surface of activated T-lymphocytes (34).

Interestingly, unlike the original study describing intracellular complement activation in T cells (63), this study with dendrimers demonstrated that the mechanism underlying nanoparticle-mediated intracellular complement activation involves membrane damage and does not induce substantial changes in cell functionality as was assessed by cytokine production in and proliferative responses of leukocytes (34). Functional consequences of dendrimer-mediated intracellular complement activation remain largely unknown. However, complement split product deposition on lymphocyte surfaces may represent a process of so-called self-opsonization, which nanoparticle-damaged cells use to alarm other cells about the presence of danger. Further investigation is required to determine whether the cell surface-exposed intracellular complement system represents another DAMP contributing to immunity. It also remains unknown whether intracellular complement system activation by dendrimers observed *in vitro* in healthy human donor lymphocytes (34) is also responsible for the delayed-type HSRs observed in a human subject after occupational exposure to cationic PAMAM dendrimers (67). To my knowledge, our team’s study (34) represents the only currently available structure-activity relationship and mechanistic investigation of nanoparticle-mediated activation of the intracellular complement system. Therefore, more studies are needed to improve current knowledge about nanoparticle effects on intracellular complement activation and its functional consequences.

## Cytokines

The communication between various immune cells and between the immune cells and other cells in the body can be direct *via* cell-to-cell contact and indirect *via* messenger molecules. Cytokines are a large group of such messenger molecules with diverse structures and functions produced and released by cells in response to inflammatory stimuli or damage. The earliest phase of the innate immune response operates with cytokines produced by macrophages and plasmacytoid dendritic cells (DCs). Other cell types, including platelets, some T cells (mainly regulatory T cells), fibroblasts, endothelial cells, and epithelial cells, can also contribute to the cytokine response during the early phase of inflammation. During this early phase, cytokines act on the nearest cells *via* the paracrine mechanism and, upon entry into systemic circulation, send the message to cells at other locations *via* the endocrine mechanism. Cytokines can have similar, overlapping, and unique functions and stimulate the production of other cytokines and secondary messengers, which amplify the response and initiate new responses. Examples of cytokines produced in the early phase of innate immune responses include tumor necrosis factor-alpha (TNFα), interleukins (IL-1, IL-12, IL-10, IL-6, IL-15, IL-18, IL-23, and IL-27), type I interferons (IFNα and IFNβ), and chemokines (IL-8, MIP-1a, MCP-1). Cytokines coordinate innate and adaptive immune responses; some of them (e.g., IFNγ, TNF, IL-5, and IL-17) are also produced by activated T lymphocytes during the adaptive immune response. Understanding cytokine responses helps interpret the results of both safety and efficacy studies. Other aspects of nanoparticle immunocompatibility, such as the immunogenicity topic described later in this review, may provide mechanistic insight into the cytokine responses to nanomaterials due to the known role of antibodies in activating the immune cells and biochemical immune pathways such as the complement pathway.

### Beneficial cytokine responses

Activation of specific cytokines by nanotechnology carriers to direct desirable immune responses is determined by the nanoparticle composition and physicochemical properties (e.g., size, charge, shape and hydrophobicity) (68) and has been extensively studied in the field of vaccines and immunotherapies (69). For example, fibrous TiO_2_ particles with a large aspect ratio were more potent at activating NLRP3 inflammasome and promoting LPS-induced IL-1β induction than their spherical and fibrous low aspect ratio counterparts (70). In another study, smaller carbon black and TiO2 nanoparticles were more potent inducing cytokines than larger particles of the same composition and surface functionality (71). An interesting example demonstrating the importance of the cell type is the study of sheet-like zinc oxide particles that induced higher levels of TNF than their spherical counterparts in murine dendritic cells but not in macrophages (72). More examples of structure-activity relationships in nanoparticle-mediated cytokine responses are reviewed elsewhere (68).

Iron, silica, chitosan, poly(lactic,glycolic) acid (PLGA), liposomes, emulsions, virus-like particles, peptide- and poly(amino acids)-based carriers, synthetic polymers (e.g., polyethyleneimine, PEI), and DNA origami have been shown in various models to improve the antigen uptake, processing, and presentation, and result in overall better vaccine and immunotherapy performance (73–86). For example, Veneziano et al., designed virus-like particles using DNA-origami technology for presenting antigens to B-cells; in this concept, the antigens were spaced out on the origami surface at a controlled distance (25-30 nm) that allowed for the most optimal activation of the B-cell receptor (85).

Using nanoparticles, researchers were able to direct specific Th1 versus Th2 polarization and major histocompatibility complex (MHC)-restricted cytotoxic T-cell responses that traditional vaccines and therapies could not achieve (73, 86–88). Through nanoparticle-mediated regulation of inflammatory pathways and cytokine production by the cells residing in the tumor microenvironment, researchers have also been able to direct the activation status of macrophages from immunosuppressive M2 to inflammatory M1 phenotypes and thereby contribute to a better outcome of cancer therapy (89–91). Likewise, nanoparticles have been used to achieve repolarization of macrophages from M1 to M2 phenotype to benefit therapy of autoinflammatory and inflammation-mediated neurodegenerative conditions (92–95). Besides inducing desirable host cytokine response supportive of either M2/M1 or M1/M2 repolarization, nanoparticles have been successfully used to deliver cytokines (such as IL-4 and TNFα) that, upon release from a nanocarrier, triggered desirable responses without toxicity to the host (16, 93, 96, 97). Nanoparticle-mediated delivery of TNFα tested in phase I clinical trials demonstrated that, unlike free cytokine, nanoparticle-bound TNFα does not induce a systemic inflammatory response and is also not immunogenic (16).

Other examples of beneficial cytokine response to nanoparticles and nanoparticle-formulated drugs include a recent study of CpG oligonucleotides delivered using particle replication in non-wetting templates (PRINT) nanoparticles. This concept resulted in particle accumulation in the lungs, where local cytokine response to delivered CpG oligonucleotides resulted in a reduction in the tumor size (98). Unlike free oligonucleotides, the PRINT nanoparticle-formulated CpG oligos did not elicit a systemic cytokine response (98). In another study, local application of doxycycline-loaded PLGA nanoparticles in the oral cavity resulted in an induction of anti-inflammatory (IL-10) and reduction in pro-inflammatory (IL-8, IL-6, IL-17, and IFNγ) cytokines, which contributed to resolving inflammation in patients with type 2 diabetes-associated periodontitis (99). Chitosan/polyglutamic-acid-formulated interferon-gamma induced the secretion of IL-12, IL-6, and TNFα, which modified the tumor microenvironment such that the invasion of colorectal cancer cells was hampered (100).

### Overt cytokine responses

In contrast to studies discussed above, an overt production of inflammatory cytokines in response to systemically administered nanoparticles has also been described for certain nanoformulations. For example, adverse immune-mediated reactions to liposomal microRNA formulation MRX34 were so severe that they led to four patient deaths and subsequent discontinuation of the clinical trial (101). The same study reported that the toxicity could be managed using immunosuppressive therapy with dexamethasone (101). Another lipid-based nanoparticle formulation of siRNA, ONPATTRO, resulted in IRs in more than 20% of patients. This response was not attributed to cytokines and, in one case, was due to the complement activation (102). The mechanism underlying these reactions in other patients remains unknown. These studies emphasize the importance of considering each nanoformulation in the context of the intended route of administration and indication and conducting extensive physicochemical characterization along with immunotoxicity assessment for the nanocarrier, API, and a final formulation containing both components.

### Cytokine responses to nucleic acid nanoparticles

Unlike traditional therapeutic nucleic acids (TNA) such as siRNA, anti-sense DNA oligonucleotides, and CpG oligonucleotides, nucleic acid nanoparticles (NANPs) are immunoquiescent in that adding these particles to immune cells does not result in a cytokine response (103). However, cytokine response to NANPs can be observed after they are delivered to immune cells using a lipid carrier (e.g., lipofectamine 2000). Earlier studies demonstrated that NANPs, after complexation with a lipid-based carrier, are internalized *via* Scavenger Receptor A-mediated phagocytosis, and this uptake culminates with the production of type I and type III interferons (103). Another remarkable difference between NANPs and TNA is that endosomal TLR7, but not TLR3 or TLR9, triggers the interferon response to RNA and DNA NANPs (104).

The expression of TLR7 is abundant in airways, and the activation of this innate immune receptor has a bronchodilating effect, decreases allergy-mediating Th2 responses, eosinophilic inflammation, and goblet-cell hyperplasia that make it a therapeutic target in asthma (105). Since the activation of TLR7 pathway inhibits viral replication in lungs and reduces airway hyperreactivity triggered by viral infections, synthetic TLR7 agonists [e.g., imiquimod (R837), resiquimod (R848), and 8-hydroxyadenine derivatives] have also been investigated as antiviral drugs (105). Collectively, the existing knowledge of targeting TLR7 for therapeutic indications opens the opportunity for NANPs to be used as antiviral and anti-asthmatic drugs.

While the initial studies are encouraging, more research is needed to fully evaluate the safety of NANP-mediated TLR7 activation because a recent study provided the first causation link between TLR7 activation and systemic lupus erythematosus (SLE), an autoimmune disorder (106), which is in line with the earlier clinical observation of TLR7-agonist association with psoriasis, an autoinflammatory skin disorder (107).

Structure-activity relationship studies revealed that the magnitude of the interferon response to NANPs could be controlled by the type of nucleic acids used to create these particles, with RNA-based NANPs being more potent interferon inducers; three-dimensional shape, with the globular NANPs being more potent than planar and fibrous NANPs; and size, but not sequence complementarity (103). More interestingly, the spectrum of cytokine response to NANPs could be controlled by the type of delivery carrier. Particularly, when amine-terminated dendrimers were used instead of lipofectamine, pro-inflammatory cytokines IL-1, TNF, and IL-6 were observed, whereas type I and type III interferons were not (108). Therefore, both the quantity (e.g., cytokine levels) and the quality (e.g., cytokine spectrum) of the innate immune responses to NANPs can be controlled by using different carriers to deliver these materials.

An extensive discussion regarding the immunotoxicity of traditional TNAs and NANPs; the role of nanocarriers in mitigating this toxicity; and translational challenges, opportunities, and barriers due to the immunological properties of NANPs are available elsewhere (109–115).

### Trends in cytokine responses to nanomaterials

**Figure 4:**
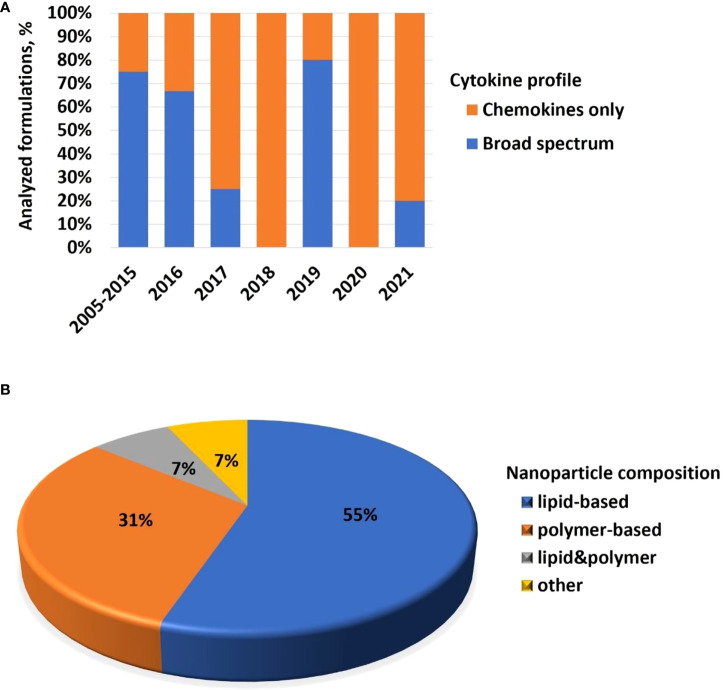
NCL assay cascade experience with cytokine analysis. Between 2005 and 2021, NCL has characterized over 450 nanotechnology formulations using assay cascade protocols (https://ncl.cancer.gov/resources/assay-cascade-protocols) that include six assays for the assessment of cytokines (ITA-10, ITA-22, ITA-23, ITA-24, ITA-25, and ITA-27). **(A)** Breakdown of formulations by cytokine profile (i.e., formulations that induced broad-spectrum cytokines versus those that exclusively induced chemokines). Percentage reflects the total number of formulations subjected to cytokine analysis. The data for 2005–2015 are pooled; during this time, IL-8 was the only chemokine on the NCL cytokine panel; other cytokines in the NCL 2005–2015 panel include TNF, IL-1β, IL-6, and IFNλ. The panel was expanded and, since 2016, includes chemokines MIP-1α, MIP-1β, MCP-1, MCP-2, and RANTES, in addition to the IL-8. Other cytokines in the extended panel are TNF, IL-1α, IL-1β, IL-2, IL-4, IL-5, IL-6, IL-7, IL-10, IL-12, IL-13, IL-15, IL-17, IL-22, IL-23, IL-27, IFNγ, IFNα, IFNβ, and IFNλ. “Broad-spectrum” refers to all or any combination of these cytokines where the combination includes cytokines of different functional types (e.g., pro-inflammatory and chemokines; pro-inflammatory and interferons; interferons and chemokines, or all of the above). Chemokines only refer to formulations that induce all or any chemokines in the absence of other functional cytokine types. **(B)** Breakdown of formulations that exclusively induce chemokines by nanoparticle composition. Most of the chemokine-inducing formulations are lipid-based, polymer-based, or contain both lipids and polymers either in the nanoparticle core or as the excipient or both. NCL, Nanotechnology Characterization Laboratory; ITA, immunotoxicity assay.

Cytokines are commonly used in preclinical studies as biomarkers of inflammation. Previously, NCL reported an interesting trend showing that lipid-based nanomaterials analyzed in the NCL standardized assay cascade between 2005 and 2015 induced chemokine IL-8 without inducing other pro-inflammatory cytokines such as TNF, IL-1, and IL-6 (32). The data were acquired using in-house developed single-plex ELISAs and several commercial multiplex platforms, including Meso Scale Discovery, BD Biosciences Cytometric Bead Array, Rules-Based Medicine MAP, and Bender MedSystems Flocytomix Multiplex Kit, and showed comparable results. In 2016, NCL switched to using chemiluminescent multiplex cytokine panels by Quansys Biosciences; these new custom multiplex assays cover 29 cytokines, including several chemokines (IL-6, MCP-1, MCP-2, MIP-1α, MIP-1β, and RANTES). During the past five years, the NCL assay cascade detected nanoformulations that induced a broad spectrum of cytokines and continued observing a trend in nanoformulations that exclusively induce chemokines, i.e., without other pro-inflammatory cytokines (**Figure 4A**). Nanoparticle composition analysis reveals that most concepts inducing chemokines are made of polymers, lipids, or containing both polymers and lipids, either as core nanoparticle carriers or excipients in the formulation (**Figure 4B**). Formulations inducing a broad spectrum of cytokines are often those that contain another cytokine as either API or targeting moiety, a TLR agonist as an adjuvant, or CpG oligonucleotide(s) as either an API or structural component of the nanoparticle.

## Coagulation system

The coagulation system’s two main components are platelets and the plasma coagulation system.

Platelets, also known as thrombocytes, are the smallest among peripheral blood cells (116). The main role of these cells is to maintain hemostasis. Physical damage to blood vessels and inflammation are among the factors that activate platelets and promote their aggregation (116). The contribution of activated platelets to IRs was described in patients undergoing therapy with a perioperative neuromuscular blocking agent and in a humanized mouse model of IgG-dependent anaphylaxis (117). Earlier studies demonstrated that nanoparticle size, charge, and density of surface functional groups determine nanoparticle interaction with platelets (118–121). For example, PAMAM and triazine dendrimers with cationic surface moieties (amine or guanidine) activated platelets and resulted in platelet aggregation; this activity was size-dependent in that larger particles were more potent than smaller particles with the same surface functionality (119). In contrast to amine-terminated PAMAM dendrimers, particles with hydroxy- or carboxy-functionalized surfaces did not activate platelets regardless of the particle size (119). PAMAM dendrimers were more potent at activating platelets than triazine dendrimers of equivalent size and surface charge (121).

It has also been demonstrated that traditional sterilization methods such as gamma irradiation and autoclaving may change nanoparticle surfaces so that the particles become pro-thrombogenic and activate platelets (122). However, the contribution of platelets to IRs in response to nanoparticles has not yet been fully investigated.

The coagulation factor family is a group of thirteen proteins that, like the complement system, are organized in a proteolytic cascade. When analyzed under *in vitro* conditions, this cascade can be divided into three pathways: an extrinsic (prothrombin time [PT]) pathway, an intrinsic (activated partial thromboplastin time [aPTT]) pathway, and a common (thrombin time) pathway. Nanoparticle interaction with plasma coagulation depends on particle composition, surface functionalization, and size. For example, amine-terminated polystyrene nanoparticles inhibited plasma coagulation by depleting plasma coagulation factors VII and IX (123). This property was size-dependent in that smaller nanoparticles were more effective than their larger counterparts (123). Surface functional groups significantly contributed to the nanoparticle interaction with the coagulation pathway in that polystyrene nanoparticles with a negatively-charged surface coating activated the intrinsic pathway; this property was also size-dependent, with large particles being more effective than their smaller counterparts (123). In contrast, anionic liposomes inhibited plasma coagulation *via* interaction with coagulation factors XII and XI (124).

**Figure 5:**
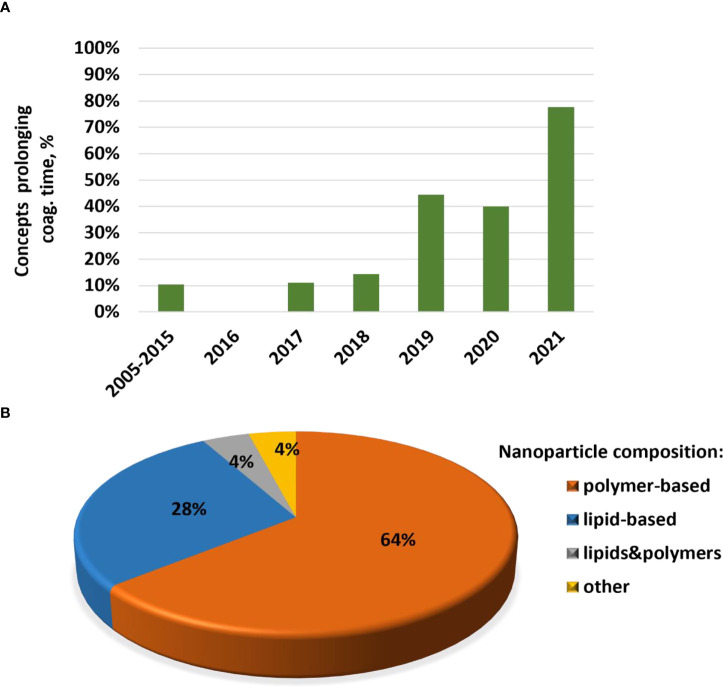
NCL assay cascade experiences with nanoparticle effects on blood coagulation. NCL has characterized more than 450 nanotechnology formulations using assay cascade protocols (https://ncl.cancer.gov/resources/assay-cascade-protocols) that include two assays for the assessment of the coagulation system (ITA-2 and ITA-12). Shown on the graph is a proportion of formulations that induced prolongation of plasma coagulation time in the NCL assay ITA-12 **(A)** and breakdown by nanoparticle composition of formulations resulting in APTT prolongation, a feature shared with traditional blood-thinning agent heparin **(B)**. Most of these concepts are polymer-based, contain lipids, or both lipids and polymers either as the core nanoparticle or excipient or both. NCL, Nanotechnology Characterization Laboratory; ITA, immunotoxicity assay.

The number of concepts characterized in the NCL assay cascade and affecting coagulation is growing with the increasing general trend of using polymer-based drug delivery systems and prodrugs (**Figure 5A**). Most of the particles affecting coagulation pathways contain polymers as a part of the carrier or as an excipient (**Figure 5B**). Common features these polymers share with traditional anti-coagulant heparin are that these polymers are polar, long, charged, and hydrophilic. This observation deserves attention for several reasons. First, because many tumors have prothrombogenic properties (125), delivering cancer therapeutics using nanotechnology platforms with anti-coagulant properties may have a collateral benefit for cancer therapy. Second, it has been demonstrated that due to its polyanionic nature, heparin binds to various proteins (126). This property contributes to heparin’s biological effects beyond blood coagulation. Particularly, heparin inhibits viral infection by competing with the virus for binding sites on target cells (127). The S1 subunit of the SARS-CoV-2 spike protein containing a receptor-binding domain was shown to bind to heparin (128). Moreover, heparin antagonizes histones released from damaged cells, thereby reducing endothelial injury during viral infection (129, 130). Therefore, I hypothesize that nanotechnology platforms with heparin-like behavior, when used for the delivery of SARS-CoV-2 therapeutics, may have collateral benefits (like that of heparin) by inhibiting viral interaction with cellular receptors and antagonizing histone-release-mediated endothelial injury.

## Immunogenicity

One of the consequences of immunogenicity significant for therapeutic products is the formation of anti-drug antibodies (ADA). The ADA can increase or decrease the product’s efficacy, cause alterations in the drug’s pharmacokinetics (PK), accelerate the drug clearance, and mediate systemic and local antibody-mediated toxicities such as anaphylaxis, HSR, kidney toxicity, and neutralization of non-redundant endogenous proteins with overlapping epitopes (131). The frequency of ADA occurrence and their clinical impact anti-correlate in that binding antibodies are the most frequent but have the least clinical impact, whereas neutralizing cross-reacting antibodies are the least frequent but have the most clinical impact. Therefore, detection of ADA and understanding their functional type (e.g., binding, PK-altering, neutralizing, HSR-causing, cross-reacting neutralizing) and isotype (e.g., IgM, IgG, IgE) are recommended by the US Food and Drug Administration for certain drug products (e.g., protein, antibody, and peptide-containing products including nanotechnology concepts) (132). Understanding the functional type of the ADA helps to estimate the risk of adverse events and their severity in the context of PK, safety, and efficacy studies. Knowing the ADA isotype provides a mechanistic insight; for example, IgE is associated with true allergy, whereas IgM and IgG are known for their ability to mediate complement activation and CARPA, as detailed in the complement section above.

Nanoparticle immunogenicity has been extensively studied using fullerenes, dendrimers, and liposomes. These studies demonstrated that nanoparticles are poor antigens and do not induce antibody responses even in the presence of potent adjuvants. For example, C60 fullerene derivatives in the presence of Freund adjuvant did not induce generation-fullerene-specific antibodies (133). However, conjugation of nanoparticles to proteins and/or administration in the presence of microbial ligands that activate toll-like receptors (TLRs) resulted in the formation of particle-specific antibodies. For example, C60 fullerenes derivatives conjugated to thyroglobulin administered in the presence of an adjuvant resulted in generation-fullerene-specific antibodies (134–136). Interestingly, C60 fullerene-specific antibodies reacted to the core and not to the terminal groups (136) and cross-reacted with C70 fullerenes and single-wall carbon nanotubes (134, 137).

Most importantly, unconjugated fullerenes, even in the presence of Freud adjuvants, were not immunogenic (133). Similar results were obtained with PAMAM dendrimers (138, 139). Dendrimer conjugation to a protein (hIL-3 or BSA) resulted in the formation of a dendrimer-specific antibody response (139). The induced antibodies reacted with dendrimer surface groups (139). Collectively these studies indicated that nanoparticles behave as haptens and that both T and B lymphocytes are involved in the immunogenicity of protein-conjugated nanomaterials.

Like fullerenes and dendrimers, liposomes alone were not immunogenic (140); however, in contrast to fullerenes and dendrimers, liposomes induced antibodies in the presence of TLR4 agonist, lipid A, which was used as an adjuvant (140–142). Pre-existing (naturally occurring) antibodies to liposome components such as phosphatidylcholine (PC), cholesterol (Chol), and dicetyl phosphate (DCP) were found in human blood (143). The mechanism underlying the formation of these antibodies is not well understood but potentially involves a prior exposure to these lipids coinciding with or related to infectious agents supplying TLR ligands as adjuvants. For example, in an experimental rabbit model, Trypanosoma rhodesiense infection led to the formation of antibodies specific to several lipids, including PC, PI, PIP, and Chol; these lipids were also detected in the pathogen used in this animal model (144). Immunization of immunologically competent but not athymic mice with liposomes and an adjuvant resulted in a liposome-specific IgM response; this finding pointed to the thymus-independent mechanism (145). Interestingly, liposome-specific antibodies also recognized phospholipids, DNA, and lipoteichoic acids (141).

Recently, the immunogenicity of hydrophilic polymer coating, particularly that of PEG, on nanoparticle surfaces became a hot topic due to the contribution of these antibodies to infusion reactions and HSRs to nanoformulations, as was discussed above in the complement section. The original intention of including PEG and other hydrophilic polymers on the particle surface was to improve nanoparticle solubility and shield them from clearance by the mononuclear phagocytic system. It was expected that extended circulation time and decreased clearance would also prevent the immunogenicity of both the particles and their therapeutic payload. Surprisingly to many researchers, PEG itself was found to be immunogenic, and various antibodies, including IgM, IgG, and IgE, specific to this polymer, were described in the blood of healthy individuals and patients treated with PEGylated or PEG-containing products (146–148). Anti-PEG IgG and IgM were primarily reviewed in the literature in the context of CARPA because antibody-antigen complexes trigger activation of the classical pathway of complement (47); these antibodies were also shown to induce premature drug release from and reduce the therapeutic efficacy of PEGylated liposomes, underline accelerated blood clearance of PEGylated products, and alter biodistribution and mobility in the mucus of PEGylated nanoparticles (149–151). Anti-PEG IgEs correlated with immediate-type HSRs (true allergy) to PEGylated products (147, 148). Importantly, anti-PEG antibodies cross-reacted with polysorbate and were found to be responsible for allergic reactions to polysorbate-containing products (147). Likewise, in another study, anti-PEG antibodies cross-reacted with other C-C-O-containing polymers, including polypropylene glycol, polyethyleneimine, and polytetramethylene ether glycol (152). The mechanism underlying PEG immunogenicity is not completely understood, but two recent reviews have discussed the application of general knowledge regarding T-independent antigens to PEG immunogenicity through the passive immunization resulting from environmental exposure and food (153, 154). Interestingly, two recent reports demonstrated anti-PEG IgG and IgM induction *via* active immunization with mRNA-PEG-LNPs in a pig model (155) and humans (156).

For many years, the hydrophilic nature of PEG made some scientists doubt the existence of anti-PEG antibodies and suggested that the unspecific antibodies are cross-reacting with ELISA components. However, structural investigation of the antibody-PEG interaction (157), along with studies linking the presence of these antibodies to HSR (47, 147, 148, 155) and premature drug release (60, 158), softened these doubts. Additional studies investigating the crystal structure of PEG-anti-PEG antibody complexes will further improve the understanding of antibody interactions with hydrophilic polymers and are urgently needed.

These unexpected but quickly expanding findings prompted many researchers to reconsider PEG use in nanomedicine and promoted the investigation of other polymers as PEG alternatives with the hope of overcoming the problem of PEG immunogenicity. Despite initially exciting findings of many such alternatives to improve solubility and increase circulation time of modified nanoparticles, they also discovered immunogenicity of these polymers, very much like earlier studies of PEG. More details about the immunogenicity of PEG alternatives (e.g., polyvinyl pyrrolidone and polyglutamic acid) and other immunological responses to polymers (e.g., heparin, polyoxazoline, and polycarboxybetaine, to name a few) used in pharmaceutical products and nanomedicines have been reviewed in detail elsewhere (154). Overall, it was concluded that no ideal PEG alternative exists; immunogenicity, allergy, and HSRs to various PEG alternatives are common. Moreover, thorough studies of immunological properties of PEG alternatives both alone and in the context of the whole product, which may contain nanoparticle carriers, APIs (e.g., protein, antibody, therapeutic nucleic acid, and small molecule), and excipients appear to be key to understanding immune-mediated reactions to this product and designing safe and effective formulations.

## Immunosuppression

Immunosuppression is a condition in which an individual’s immune response is lowered. It can result from genetic mutations affecting receptors, adaptor proteins, or transcription factors involved in the normal innate and adaptive immunity (159, 160). For example, the mutation in IRAK4 increases susceptibility to infections (161, 162). Immunosuppression may also be due to environmental factors (e.g., xenobiotics) and certain types of drug products (163–165). Drug-mediated immunosuppression can be desirable [i.e., used to suppress a known overt activation of the immune system to prevent host damage (e.g., dexamethasone helps to prevent damaging effects of cytokine storm during bacterial or viral sepsis, rejection of organ transplant, or for suppressing an autoimmune response)] (164, 166) or adverse(i.e., when it is not intended but weakens the host’s response to microbes and cancer [e.g., chemo and radiation therapy target cancer cells but also damage nontarget immune cells]) (163, 165, 167). Drugs intended to modulate the function of immune cells may also cause adverse immunosuppression. For example, cyclosporin, intended to prevent transplant rejection, when taken for a long time, may also increase the risk of bacterial and viral infections (168). To reduce the negative consequences of immunosuppressive therapies, vaccination and prophylactic anti-microbial therapies are often considered for patients receiving such drugs (167, 169).

**Figure 6:**
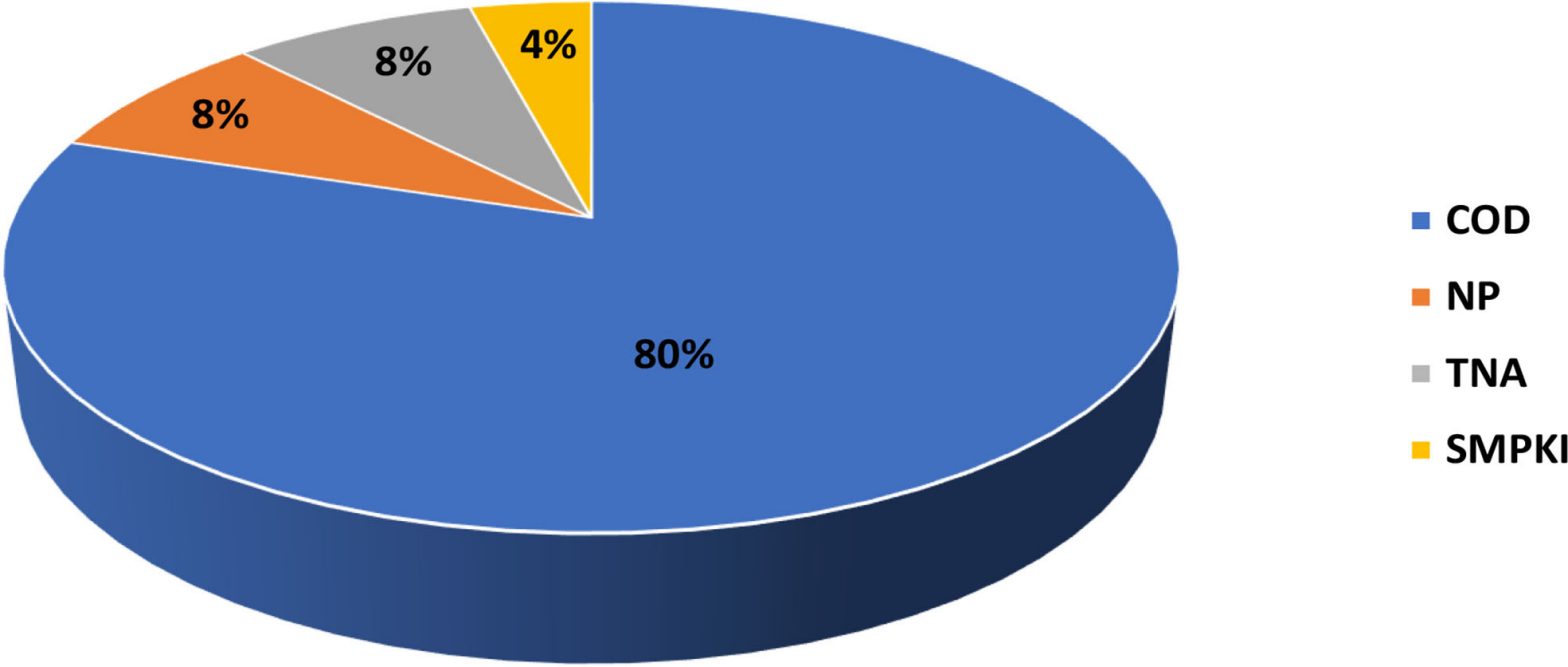
Immunosuppressive properties of nanotechnology formulations characterized at NCL. NCL has characterized more than 450 nanotechnology formulations using assay cascade protocols (https://ncl.cancer.gov/resources/assay-cascade-protocols) that include two assays for the assessment of immunosuppression (ITA-6 and ITA-18). Shown on the graph is a proportion of formulations that were immunosuppressive in these *in vitro* assays due to either API or carrier. The immunosuppressive properties attributed to APIs included those due to small molecules: cytotoxic oncology drugs (COD), therapeutic nucleic acids (TNA), small-molecule protein kinase inhibitor (SMPKI), or nanoparticle platform (NP). API, active pharmaceutical ingredient; NCL, Nanotechnology Characterization Laboratory; ITA, immunotoxicity assay.

Cytotoxic oncology drugs intend to stop cancer cell proliferation but also affect lymphocytes, thereby decreasing lymphocyte-mediated immune responses (170). When such APIs are delivered using nanotechnology platforms, final formulations may inherit the immunosuppressive properties of APIs. For example, among nanotechnology-formulated drugs that were characterized by NCL between 2005 and 2020, the majority (92%) were immunosuppressive due to the APIs, while only a small proportion (8%) was due to the nanocarrier (**Figure 6**).

Below, I review some examples of drug-mediated immunosuppression due to bone marrow (BM) and blood lymphocyte inhibition and discuss whether and how nanotechnology platforms influence this toxicity. Whenever available, I will also discuss the immunosuppressive properties of nanocarriers themselves.

### Bone marrow

Nanocarriers may influence drug distribution to BM, thereby diminishing or enhancing the drug-mediated toxicity. For example, in one early study, DXR, formulated on polyisobutyl (PIBCA)- and polyisohexyl (PIHCA)- cyanoacrylate nanoparticles, demonstrated differential distribution and toxicity (171). DXR-PIBCA suppressed the formation of granulocyte–macrophage progenitor (CFU-GM) after i.v. injection in mice, and this toxicity was comparable to the effect of free DXR; however, at an equivalent drug dose, DXR-PIHCA were more immunosuppressive. Similar effects were observed on spleen cells with a decrease in granulocytes and lymphocytes being more pronounced with DXR-PICHA formulation. Both PIBCA and PICHA carriers alone were not toxic. The authors linked greater toxicity of PICHA- versus PIBCA-formulated DXR to the more significant accumulation of PICHA-formulated drug in BM and spleen; however, the mechanisms underlying such differential biodistribution were not identified but were hypothesized to relate to different rates of opsonization that determined the greater uptake of nanoparticle-formulated drug by phagocytic cells in target organs (171). Another study found that the uptake of unfunctionalized- and citrate-stabilized IONPs by BM cells *in vitro* exceeded the uptake of iron citrate used as a control. Greater uptake, however, did not influence cell viability and expression of surface markers (172). Unlike PICHA and PIBCA nanoparticles in the study by Gibaud et al. (171), IONPs were not loaded with an oncology drug; therefore, the lack of difference in toxicity may be explained by the generally biocompatible nature of the iron oxide platform (172). Provided the greater accumulation of IONPs in BM remains after the drug conjugation, I expect a similar increase in the BM cytotoxicity of the drug-formulated IONPs. Apart from biodistribution, drug-mediated myelosuppression may be influenced by the rates of drug release from nanocarriers. For example, docetaxel conjugated to solid LNPs was less myelosuppressive than docetaxel at equivalent concentrations *in vitro* in a colony-forming unit assay (173).

Accumulation of some nanoparticles in BM resulted in myelosuppressive effects due to particle-mediated apoptosis and hypoplasia. For example, intraperitoneal administration of aluminum oxide nanoparticles to mice decreased total and differential BM counts and altered erythropoiesis (174). The same study also reported myeloid hyperplasia due to the inflammation-associated increase in neutrophil precursors. The damaging effects of aluminum oxide nanoparticles on BM were neutralized by co-treatment with curcumin nanoparticles; the protective effects of nanocurcumin were attributed to its anti-inflammatory properties (174).

### Blood lymphocytes

Suppression of lymphocyte function may occur due to either immunosuppressive drug payload or nanocarrier per se. Examples of drug-mediated immunosuppression include PLGA-betamethasone and nanoalbumin-paclitaxel (Abraxane), among others (175–177). Drug-mediated immunosuppression is common for nanotechnology concepts delivering cytotoxic oncology drugs.

Inhalation of carbon nanotubes suppressed B-lymphocytes’ function *via* TGFβ produced by alveolar macrophages (178). An interesting example is the iron-oxide formulation Feraheme (ferumoxytol) used for iron deficiency in chronic kidney disease patients. While adverse effects of this formulation commonly discussed in the literature include HSRs and CARPA, both attributed to the dextran coating on the surface of IONPs (179–181), this formulation was also found to be immunosuppressive and inhibited human T-cell function *in vitro* (182) and *in vivo* (183). Feraheme inhibited cytokine secretion and antigen-induced proliferation of T cells by inducing mitochondrial oxidative stress (182). Interestingly, Th17 function inhibition and IL-17 secretion by these cells in response to Feraheme *in vitro* (182) was suggested for potential use in relieving inflammation leading to psoriatic skin lesions *in vivo*. In a subsequent study, using a mouse model of chemically induced psoriasis, topical application of Feraheme was almost as effective as hydrocortisone in reducing skin inflammation (183). Another study demonstrated that Feraheme’s ability to suppress myeloid-derived suppressor cells has beneficial effects on recovery from endotoxin tolerance following sepsis (184).

## Available methods and models to study immunotoxicity

This section will discuss assays for assessing nanoparticle effects on the integrity and function of immune cells commonly used in preclinical research. Nanoparticles must undergo analysis for sterility and contamination with innate immunity-modulating impurities prior to *in vitro* and *in vivo* immunotoxicity studies since microbes and their components (e.g., endotoxin, beta-glucans, and CpG DNA) may confound the results of such studies (185). Challenges with endotoxin and beta-glucans detection in nanomaterials from NCL’s experience have been described earlier (19, 32, 186–190). Reports on methodologies for endotoxin detection in nanomaterials from other laboratories are also available (191–196).

### *In vitro* methods

#### Hemolysis

An *in vitro* hemolysis test is conducted to assess nanoparticles’ effects on the integrity of red blood cells. Various experimental protocols for hemolysis studies using human and animal blood are available and have been discussed in more detail elsewhere (197). The *in vitro* method that incubates human whole blood with test nanomaterials and then detects plasma-free hemoglobin (198) shows a good *in vitro*-*in vivo* correlation. As reported earlier, as low as 5% of hemolysis detected by this method *in vitro* correlates with hemoglobin and hematocrit alterations *in vivo* (199). Nanoparticles that are found hemolytic in the NCL assay cascade possess common structural properties, including cationic surface moieties and the presence of detergents and detergent-like molecules as APIs or excipients.

#### Complement activation

This assay is used to assess nanoparticles’ propensity of causing CARPA. Several formats of this method exist. One of the commonly used methods employs plasma or serum from human donors or animals, which, after exposure to test nanomaterials or controls, are analyzed by western blot or ELISA for the presence of the complement split products (C3a, iC3b, C4a, C5a, Bb, and/or sC5b-9) (200, 201). Szebeni’s laboratory established good *in vitro*-*in vivo* correlation for this method both in the human and animal (pig, rats) matrix (202–205).

When the *in vitro* complement activation assay is used for nanoparticle characterization, it is essential to consider both inter- and intra-species variability in complement activation, which may influence the assay sensitivity and overall conclusions. For example, when mouse plasma from several strains (Balb/c, CD-1, C3H/HeN, C57BL/6, and DBA1) was used as a matrix to study complement activation by liposomal amphotericin (Ambisome), the highest complement activation was observed in the plasma of Balb/c and CD-1 mice, whereas the lowest activation was seen in plasma of C57BL/6 mice; the activation in plasma of other strains was moderate (206). Interestingly, Balb/c and CD-1 mice are known for their Th-2 bias and preferred for sensitization studies, whereas C57BL/6 mice are Th-1-biased animals and are preferred in vaccine and autoimmunity studies (207). Another interesting observation is the difference in magnitude of complement activation by various agents. For example, human, but not mouse, plasma is susceptible to the complement activation by cobra venom factor (CVF) that is commonly used as a positive control for *in vitro* studies; however, the magnitude of the complement activation by Ambisome is comparable between human and mouse plasma (206). Another topic commonly discussed in the context of *in vitro* complement activation assay is the anticoagulant used to generate blood plasma. Hirudin is generally agreed as the best anticoagulant (208–210); however, this anticoagulant is not widely available. In the absence of hirudin, sodium citrate or EDTA-anticoagulated plasma can be used as long as veronal buffer is also used to supply divalent cations required for the complement activation.

#### Coagulation system

When analyzing the coagulation system in preclinical studies, it is essential to recognize that all components of this system are closely connected *via* positive and negative regulation loops. Plasma coagulation controls the activity of the zymogen prothrombin and a serine protease thrombin; Factor IIa (α-thrombin) is a final product of prothrombin activation that results in platelet activation and fibrinogen-to-fibrin conversion. Thrombin activates transamidase Factor XIIIa, which stabilizes the fibrin network with activated platelets, thereby forming a blood clot. Positive feedback of thrombin activation includes the activation of coagulation factors XI, IX, V, and VIII. The negative feedback controls the thrombin activity: thrombin binding to thrombomodulin expressed on the surface of endothelial cells activates protein C and stops further procoagulant activity. Activated protein C and its cofactor protein S activate proteolytic degradation of activated coagulation factors Va and VIIIa, which function to accelerate the thrombin-generation pathway. Thrombin also activates complement, leukocytes, and other cell types. Activated by thrombin and complement cells contribute to the plasma coagulation by producing cytokines and expressing the phospholipid-protein procoagulant activity complex. This complex initiates plasma coagulation by activating coagulation factor VII.

Nanoparticle effects on the coagulation system are commonly assessed *in vitro* using platelet aggregation, plasma coagulation, and leukocyte procoagulant activity assays (211). Platelet aggregation can be accessed using light transmission aggregometry and direct counting of single (unaggregated) platelets. Common plasma coagulation assays include APTT, prothrombin time (PT), thrombin time (TT), and reptilase time (RT) assays. The APTT assay assesses functionality of factors XII, XI, IX, VIII, X, V, II; the PT assay does so for factors VII, X, V and II; TT and RT assess the role of fibrinogen. Alteration in the fibrinogen conversion to fibrin can also be detected in all of these assays. The PT assay is also used to access the procoagulant activity of leukocytes and endothelia cells; in this case, the cells are used instead of the Neoplastin-TM reagent to activate the plasma coagulation.

Despite their common use in nanoparticle hemocompatibility studies, abnormal results of these *in vitro* assays are often challenging to interpret due to the complex effects of nanoparticles on individual components of plasma coagulation, often synergistic and antagonistic effects, and generally low specificity or sensitivity for discrimination between individual pathways of nanoparticle interactions with the coagulation system. Other methodological aspects of thromboelastography, synthetic substrate-based assays, ELISA, fibrinolytic, thrombolytic activity, and other assays for coagulation assessment have been discussed elsewhere (212).

#### Cytokines

**Figure 7:**
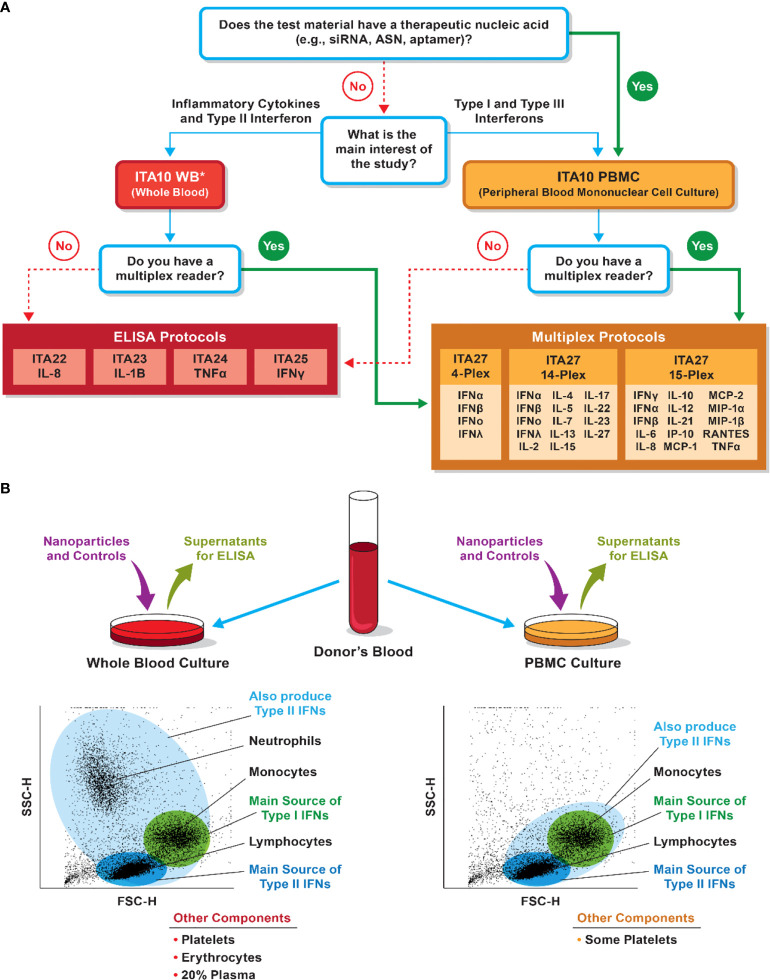
Considerations for selection of whole blood versus PBMC cultures for cytokine analysis. **(A)** NCL decision tree for model selection. The decision is influenced by the nanoparticle composition, study questions, and instrument availability. *PBMC could be used to assess pro-inflammatory cytokines and answer questions related to the risk of the cytokine storm. Whole blood, however, is a better system if type II interferon induction is of interest. **(B)** Differences in immune-cell populations between the whole-blood and PBMC cultures may influence the detection of various cytokines. Bullet points at the bottom list other cells or matrices present in the culture but not shown in the forward and side-scatter cytometry plots.

Two types of primary cell-based systems are available to cytokine researchers. They include whole blood cultures and peripheral blood mononuclear cells (PBMCs). If the cytokine of interest is expressed by cells of low abundance in the whole blood and even in PBMCs (e.g., plasmacytoid dendritic cells or γδT-cells), researchers could isolate these cells from the blood and concentrate them prior to analysis *in vitro*. Both negative and positive selection reagents are available when enrichment of a particular cell population is of interest. When such enrichment is not needed, the decision between whole blood and PBMCs could be made based on the type of cytokines one wants to detect (**Figure 7**). **Table 1** summarizes human cytokines that are commonly analyzed in preclinical and clinical studies and included in the NCL multiplex panel. The information in this table could be used to guide both study design and data interpretation.

**Table 1:** Human cytokine panel used at NCL.

Cytokine	Primary cell source	Effector target and function
IL-1α	Monocytes, DCs, macrophages, endothelial cells, hepatocytes	Endothelial cells (activation, inflammation, coagulation) Hypothalamus (fever)
IL-1β	DC, macrophages	Endothelial cells, hypothalamus (fever); liver (synthesis of acute-phase protein); Th17 (differentiation)
IL-2	T cells	T cells (activation, proliferation, and differentiation) NK cells (proliferation and activation) B cells: proliferation, antibody synthesis (*in vitro*)
IL-4	CD4+ T cells, mast cells	B cell (activation, proliferation, and differentiation)
IL-5	T cells, mast cells	B cell (isotype switching to IgE) T cells (Th2 differentiation, proliferation) Macrophages (alternative activation and inhibition of IFNγ-mediated classical activation)
IL-6	T cells, macrophages	Liver (synthesis of acute-phase protein); B cells (proliferation of antibody-producing cells); Th17 (differentiation)
IL-8	Monocytes	Neutrophil recruitment
IL-10	T cells, primarily T regs	Macrophages, DCs (inhibition of IL-12 expression, co-stimulators, and class II MHC)
IL-12	DCs, macrophages	Th1 differentiation; NK and T cells (IFNg synthesis, increased cytotoxicity)
IL-13	Th2, NKT, ILC2, mast cells	B cells (isotype switching to IgE); Epithelial cells (increased mucus production); Macrophages (alternative activation)
IL-15	Monocytes	CD8+ memory T cells (survival and proliferation) NK cells (proliferation)
IL-21	Th2, Th17	B cells (activation, proliferation, and differentiation); Tfh-cells (development); Th17 (increased generation)
IL-22	γ/δ T cells, ILC3	Epithelial cells (production of defensins, increased barrier function); hepatocytes (survival)
IL-23	Monocytes, DCs	Th17 (differentiation and expansion)
IL-27	DCs, macrophages	T cells (enhancement of Th1 and inhibition of Th17 differentiation) NK cells (IFNγ synthesis)
MIP-1α	Monocytes, macrophages	Mixed leukocyte recruitment
MIP-1β	Monocytes, macrophages	T cells, monocytes, NK recruitment
MCP-1	Monocytes, macrophages	Mixed leukocyte recruitment except for eosinophils (monocytes, T lymphocytes, NK cells, basophils, mast cells)
MCP-2	Monocytes, macrophages	Mixed leukocyte recruitment, including eosinophils (monocytes, T lymphocytes, NK cells, basophils, mast cells, and eosinophils)
RANTES	T cells	Mixed leukocytes recruitment
TNFα	NK cells, T cells, monocytes, macrophages	Endothelial cells, neutrophils (activation); hypothalamus (fever); muscle, fat (catabolism, cachexia)
IP-10	Monocytes, macrophages	Effector T-cell recruitment
IFNα (Type I)	Plasmacytoid dendritic cells (pDCs)	All cells (antiviral state, increased class I MHC); NK cells (activation)
IFNβ (Type I)	pDCs	All cells (antiviral state), increased class I MHC; NK cells (activation)
IFNω (Type I)	pDCs	All cells (antiviral state, increased class I MHC); NK cells (activation)
IFNγ (Type II)	T cells (Th1, CD8+), NK cells	Macrophages (classical activation); B cells (isotype switch to opsonizing and complement-fixing IgG subclasses); Th1 differentiation; various cells (increase in class II MHC expression, Ag processing, and presentation to T cells)
IFNλ (Type III)	pDC	DCs, neutrophils, CD4+ T cells, and B cells

This table summarizes cytokines, their origin, and their effector function. The information about these cytokines is based on references (213, 214). Knowing the primary cell source and effector/target function of the cytokines induced by nanoparticles helps to identify cell types affected by the analyzed formulations and to predict the biological effect(s) of such induction. This information aids both safety and efficacy studies.

#### Leukocyte proliferation

Leukocytes can be activated by mitogens such as plant lectin phytohemagglutinin (PHA) for T cells and lipopolysaccharide for B cells. Antigen-specific lymphocytes can also proliferate in response to their cognate antigens (e.g., flu antigens). Proliferating cell expansion can be detected by several commercially available kits and reagents with (3-(4,5-dimethylthiazol-2-yl)-2,5-diphenyltetrazolium bromide (MTT), bromodeoxyuridine, (BrdU) and carboxyfluorescein diacetate succinimidyl ester (CFSC), being broadly used (215, 216). BrdU is preferable as it detects proliferating cells that incorporate this molecule into their DNA. While increased cell viability detected by the MTT assay generally reflects on the number of viable and expanded cells, the MTT signal may also go up when nanoparticles do not induce proliferation but rather improve cell viability by supplying nutrients into the culture medium; for example, nanoformulations containing sucrose are often seen as those increasing the MTT signal. However, such an increase in the cell viability is usually minor and can be easily distinguished from a true mitogenic effect. Nanoparticles may activate the cells and promote proliferation induced by traditional stimuli, and this property is used to estimate their mitogenic activity. Some nanoparticles, especially those formulated to deliver cytotoxic drugs, inhibit or suppress the proliferation induced by mitogens (e.g., PHA-M) or antigens (e.g., flu antigen). Identification of nanomaterials’ ability to suppress mitogen- or antigen-induced proliferation is commonly used to identify immunosuppression (216). A popular *in vitro* assay that is a surrogate of the *in vivo* T-cell-dependent antibody response (TDAR) for immunosuppression screening is the human lymphocyte activation (HuLa) test that employs PBMCs of healthy donors immunized with the current-year flu vaccine. The HuLa assay was initially developed and validated across immunosuppressive drugs with various mechanisms of action and showed consistent performance (217, 218). This method is also instrumental in identifying nanoparticles with immunosuppressive properties (216).

#### CFU-GM

Hematopoietic stem cells present in the BM proliferate and differentiate to form so-called colony-forming units (CFU). Depending on the growth factors present in the culture medium, these CFU can be of different cell linage. CFU-GM, for example, assesses the formation of granulocytes and macrophages; CFU-E, erythrocytes; and CFU-GEMM, erythroid and mixed myeloid cells. This method is commonly used to assess the functionality of BM stem cells and the potential effects of test substances on these cells. The method can be conducted *in vitro* and ex vivo. In the *in vitro* protocol, the BM stem cells are isolated from untreated animals or human-donor volunteers, followed by the *in vitro* treatment with nanoparticles. In the ex vivo format, the BM cells are obtained from animals exposed to nanoparticles. Although the *in vitro* method does not account for nanoparticle biodistribution, it allows for rapid identification of potentially toxic formulations and is helpful in cases when amounts of nanoparticles are limited, and the dose information is unavailable, i.e., early in preclinical development. When BM cells are cultured in a methylcellulose-based medium in the presence of SCF, IL-3, and IL-6, it results in the formation of the CFU-GM that can be enumerated. Therefore, the *in vitro* CFU-GM protocol is used to assess the myelosuppressive properties of cytotoxic oncology drugs or nanoformulations delivering these compounds. The comparison between CFU-GM in the untreated sample (the baseline) and nanoparticle-treated sample (test) allows for the identification of nanomaterials with myelosuppressive properties (219). When conducted *in vitro* using murine or human BM cells, the CFU-GM assay was also found to accurately predict a drug’s clinical maximum tolerated dose (MTD) in human patients (220, 221).

#### Phagocytic function

Phagocytes’ primary function is to engulf and eliminate foreign particles, microbes, and abnormal host cells. Drug- or xenobiotic-mediated alterations in phagocytosis may lower the host’s response to pathogens and transformed cells. Therefore, investigation of nanoparticle effects on phagocytosis is commonly included in experimental frameworks used to assess the safety of nanotechnology-based drug products. Tracking the uptake of model foreign bodies (e.g., yeast zymosan or heat-killed *E. coli*) could be done by flow cytometry or confocal microscopy; in this case, the model particulates are conjugated to a fluorescent label. When unconjugated particulates are used as model foreign bodies for monitoring phagocytic function, a luminescence-producing reagent, luminol, is used to detect their uptake by a plate-reader-based assay (222, 223).

#### NK cytotoxicity

Natural killer (NK) cells are staffed with cytoplasmic granules containing cytotoxic proteins, such as perforin and granzymes. These proteins form pores in tumor and virus-infected cells when released, thereby contributing to the innate immune response against abnormal and infected cells. Alterations in the NK cytotoxicity may impair immunity; therefore, NK cell function analysis is an integral part of immunotoxicity studies. Both model cell lines and primary NK cells are used for such studies. For example, NK92 and HepG2 cell lines are frequently used as effector and target cells, respectively; the viability of HepG2 cells in the presence of untreated or nanoparticle-treated NK92 cells can be monitored in real-time using label-free technology (224). Other experimental approaches include whole-blood and PBMC cytotoxicity assays in which CFSE-labeled K562 target cells are monitored by flow cytometry to assess the cytotoxicity of primary effector NK cells. Another flow-cytometry-based approach includes the CD107a degranulation assay, in which whole blood or PBMCs serve as the source of primary NK cells (225, 226).

### *In vivo* models

After the initial immunotoxicity assessment using general toxicity studies, specialized immune function tests can be employed to further interrogate adverse effects on the immune system. Some of these specialized immune function tests are described below. In these methods, test nanomaterials are administered as the dose level, using the dosing regimen and *via* the route of administration relevant to the intended clinical use of these materials.

#### Rabbit pyrogen test

Systemic exposure to pyrogens (i.e., fever-causing substances) results in an elevation in body temperature. As such, the rabbit pyrogen test (RPT) was established to detect fever-causing drugs and other medical products to prevent overt responses in patients. The experimental procedure involves the injection of a test material into the ear vein of a rabbit; the animal’s body temperature is monitored before the injection and three hours after the injection with 30-minute intervals. The RPT is standardized for worldwide use in the field of drug development and pharmaceutical analysis for pyrogenicity and is documented in pharmacopoeias of various countries. However, some discrepancies exist between protocols used in various countries with regards of the required number of rabbits, the acceptable initial body temperature, the determination of baseline temperature, and the decision algorithm (227–229). Historically, the RPT was used to detect endotoxin, a pyrogenic component of the cell wall of gram-negative bacteria that is a common contaminant in pharmaceutical products. However, after the discovery of the *in vitro* limulus amoebocyte lysate (LAL) assay (230, 231), the pharmaceutical community largely switched to this *in vitro* method to detect endotoxin. Later, the *in vitro* PBMC and whole-blood cytokine test, also known as monocyte activation test (MAT), has been validated as a reliable surrogate for LAL and RPT to test not only for endotoxin but for non-endotoxin pyrogens (228, 232–238). Moreover, the experience with some biotechnology-derived therapeutics demonstrated that product processing such as lyophilization may affect the ability of LAL and RPT to accurately detect endotoxin resulting in a product that passes these traditional tests but results in a fever in human patients; in contrast, incubation of the product with PBMC reliably detected “leukocytic pyrogen” produced in response to the endotoxin that was present in the product but remained undetectable by LAL and RPT (239). Currently, all methods—*in vivo* RPT and *in vitro* LAL and MAT—are used for pyrogenicity screening, though LAL remains the most popular.

#### Murine local lymph node proliferation

Guinea Pig Maximization Test, Buehler’s test, and local lymph node assay (LLNA) have been developed to test for delayed-type hypersensitivity (DTH) reactions. More recently, the local lymph node proliferation assay (LLNP) was proposed for the prediction of DTH; this method accurately predicted DTH reactions to systemically administered pharmaceuticals (240). In LLNP protocol, test materials and controls are subcutaneously injected to mice once a day for three consecutive days; next, the animals are allowed to rest for two days before intravenous administration of ^3^H-thymidine; five hours after the thymidine injection, the animals are sacrificed, and their draining lymph nodes are analysed by scintillation counting to detect thymidine incorporation into the DNA of proliferating leukocytes. An increase in the thymidine incorporation points to T-cell activation that occurs during allergic sensitization. The LLNA protocol is identical to that of LLNP except for the route of test-material administration. In the LLNA assay, the test material is topically applied to the animal’s skin; this test is applicable to nanomaterials formulated as creams or lotions. *In vitro* assays myeloid U937 skin sensitization test (U-SENS also known as MUSST) and human cell line activation test (h-CLAT) were developed as surrogates for LLNA/LLNP and showed consistent performance in interlaboratory studies (241–243). However, when applied to nanomaterials testing, the results of these *in vitro* assays do not always correlate with that of the *in vivo* LLNP studies. For example, greater rate of positive response was observed using *in vitro* methods than using *in vivo* tests with MUSST/U-SENS being more sensitive in identifying positive responses than h-CLAT (244). Therefore, the *in vitro* assays are recommended when rapid screening of multiple nanoformulations is needed, but once positive responders are identified, they need to be re-tested using an *in vivo* method.

#### T-Cell-dependent antibody response

This method is used to assess the immunosuppressive properties of a test material. The assay is conducted in mice. First, the animals are exposed to the test nanomaterials. Next, they are injected with a substance known to produce a TDAR (e.g., keyhole limpet hemocyanin). Finally, the levels of the antigen-specific IgM and IgG are assessed one and three weeks from the antigen administration (216). A decrease in the antibody titer indicates immunosuppressive properties of the test material. The results of this *in vivo* test for iron oxide formulation Feraheme correlated with the *in vitro* HuLa assay discussed above; of note, a sex-dependent difference was detected by the TDAR method (216). Inhibition of the T-cell function by Feraheme has also been confirmed both *in vitro* and *in vivo* in other models (182, 183). However, as with any study, differences may be observed between *in vitro* and *in vivo* tests for various nanomaterials. Therefore, like the strategy mentioned above for the DTH studies, every nanoformulation should be considered on a case-by-case basis; the *in vitro* method is suitable for quick screening, whereas the *in vivo* study should be considered to verify the *in vitro* findings.

#### Porcine model for CARPA

Pigs are infused or injected with nanomedicines, and hemodynamic changes are monitored in real-time, followed by ex vivo blood sample analysis for the presence of complement split products and other inflammatory mediators such as thromboxane; the model reproduces symptoms and molecular markers induced in response to various nanomedicines known to cause IRs in human patients (48, 245, 246). Clinical relevance of this animal model has been extensively discussed elsewhere (52).

#### Genetically engineered, humanized, and naturalized models

**Figure 8:**
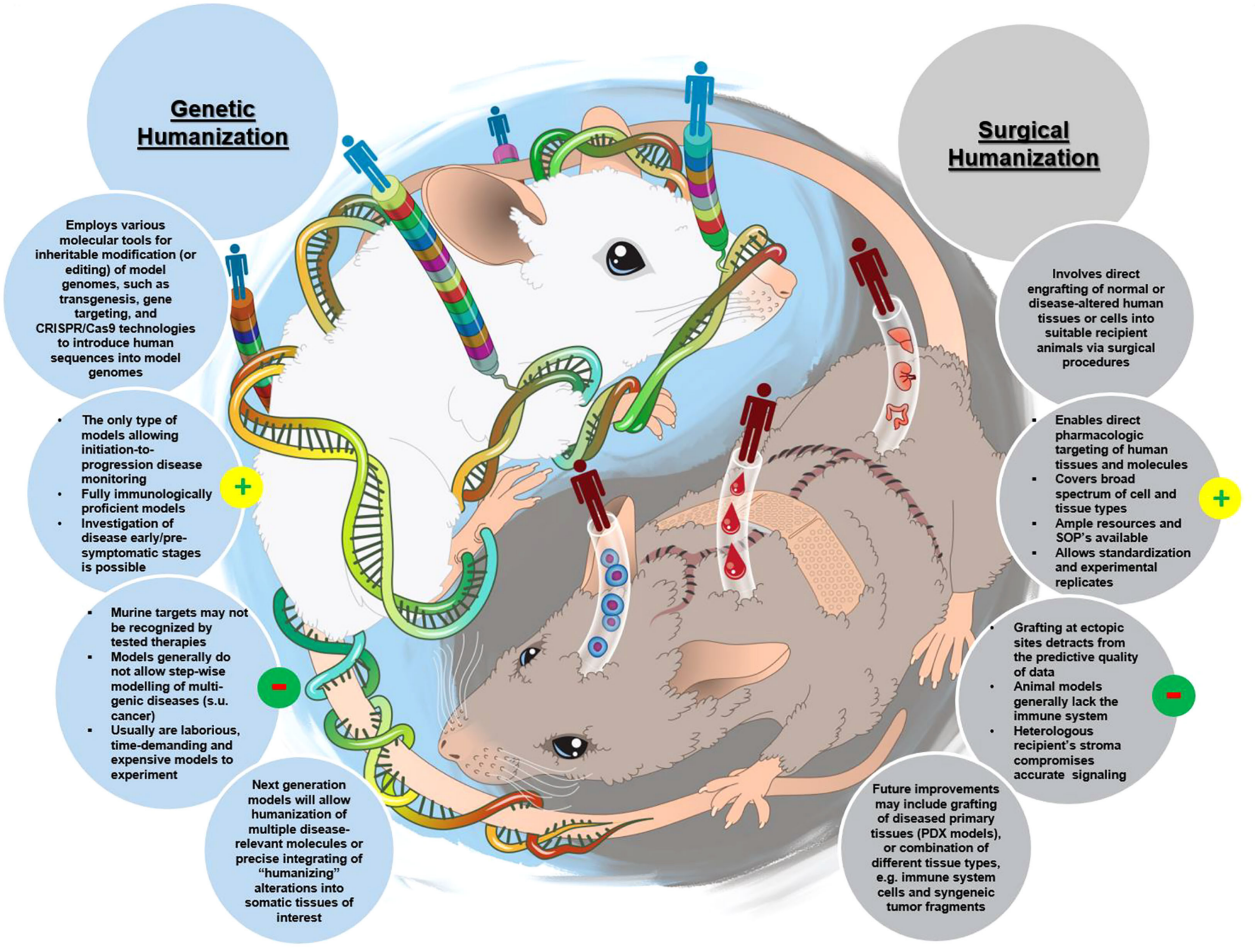
Complementary approaches in establishing humanized animal models for basic and translational research purposes. Key benefits (highlighted as yellow circle with a plus sign inside) versus shortcomings (highlighted as a green circle with a minus sign inside) of both strategies are listed, as well as anticipated future directions toward improved utility of these models for preclinical drug-assessment studies. This figure is reproduced with permission from (254).

Genetically engineered and humanized models have been developed to assess human-like immune responses in animals (247–251). Such assessment in preclinical studies is often needed when animals do not express the target for nanoparticle-formulated drugs or when drug efficacy requires immunocompetent animals. Genetically engineered mouse models (GEMMs) are ideal for studies of cancer and other diseases due to unique mechanistic insights that traditional models cannot provide; these models were reviewed in detail elsewhere (252). An example demonstrating the utility of these models in preclinical studies of nanomaterials is the Taxane-resistant GEMM strain FVB/NJ containing C3(1)SV40 T-antigen (C3Tag) transgene used to demonstrate the efficacy of PRINT nanoparticles against taxane-resistant triple-negative breast cancer (253). However, their high costs and complex logistics limit their use in research and development to specialized facilities equipped to support such models. Humanized animal models were developed by surgical transplantation of human cells or tissues, or by genetic engineering to express desired human proteins, and are more widely used in preclinical research due to their wider accessibility (**Figure 8**) (254). In one such study, PRINT nanoparticles were tested in NOD.Rag1−/−Il2rg−/− (NRG) mice, which, after irradiation, received an intrahepatic injection of CD34+ cells from human fetal liver tissues to produce human blood cells. This study found PRINT nanoparticles’ preferential uptake by human CD14+ monocytes without induction of systemic inflammation; these data in the humanized animals correlated with the *in vitro* uptake studies performed using human PBMCs (255). Another study utilized NOD/*scid*/*IL2r* common γ chain null (NSG) mice following the transfer of human PBMCs to analyze the functionality of the antigen-specific human regulatory T cells induced by PLGA nanoparticles co-delivering IL-2 and TGF-β to produce the tolerogenic response for lupus therapy (256). Similarly, NSG mice engrafted after the irradiation with human CD34+ peripheral blood stem cells derived from granulocyte colony-stimulating-factor-mobilized healthy donors were found instrumental for the *in vivo* efficacy analysis of protein subunit vaccines delivered by self-assembling protein-based nanoparticles to prevent Epstein-Barr virus infection (257).

Another interesting and thought-provoking idea for improving animal model relevance to humans is so-called “rewilding” or naturalizing the animals (258). One study demonstrated that naturalizing or rewilding animals by exposing them to the natural environment increased the maturity and diversity of lymphocytes and diversified the gut microflora (259). Graham reviewed multiple studies across several animal species, demonstrating that transitioning animals from standard husbandry conditions to a natural environment diversified the immune repertoire of the naturalized animals and suggested considering these animals for preclinical studies (258). A comparison between standard husbandry and the natural environment for C57BL/6 mice, as an example, is provided in **Table 2**. Moving preclinical studies in this direction would increase data variability, logistical challenges, and costs of such studies; however, such costs may be warranted, especially if this approach helps improve the predictability of preclinical animal studies and their relevance to humans. It would be interesting to compare biodistribution, safety, and efficacy of the same nanoformulation in the same laboratory animal strain when it is kept under standard husbandry conditions versus when it is naturalized.

**Table 2:** Differences between standard husbandry conditions and the natural environment.

Condition	Standard Husbandry	Natural Environment
Optimal temperature	21°C	29–31°C
Light/Dark Cycle	12h/12h	Varies with season
Food	Less diverse	More diverse
Physical activity	Low	High
Likelihood of exposure to microbes, parasites, and allergens	Low	High
Behavioral complexity (ability to navigate or maintain vigilance for predators)	Low	High
Leukocytes	Less mature	More mature
Immune repertoire	Less diverse	More diverse

The table summarizes various conditions for C57BL/6 mice based on reviewed literature (258). Naturalizing or rewilding the animals by exposing them to the natural environment increased the maturity and diversity of lymphocytes and diversified the gut microflora (259).

## Conclusion and future directions

After almost two decades of researching immunological properties of nanomaterials, common trends have been identified for certain nanoparticles based on their composition (e.g., polymer- and lipid-based nanomaterials induce chemokine response and prolong plasma coagulation time), surface moieties (e.g., the presence of PEG increases the risk of anti-PEG antibody-mediated responses), zeta potential (e.g., cationic materials are pro-thrombogenic and cytotoxic), shape (e.g., fibrous nanomaterials cause lysosomal rupture with subsequent activation of inflammasome), and size (e.g., large [< 300 nm] materials regardless of their surface coating are quickly eliminated by the phagocytic cells) as reviewed in this manuscript and earlier reports from NCL (32, 199, 260, 261) and other groups (22, 262–271). Knowing these trends helps prioritize safety studies and select nanoparticle platforms for formulating non-immunologically inert APIs. However, each component of nanoformulation has a role and unique properties; therefore, each nanoparticle must be considered on a case-by-case basis and in the context of APIs, excipients, route of administration, and indication.

**Figure 9:**
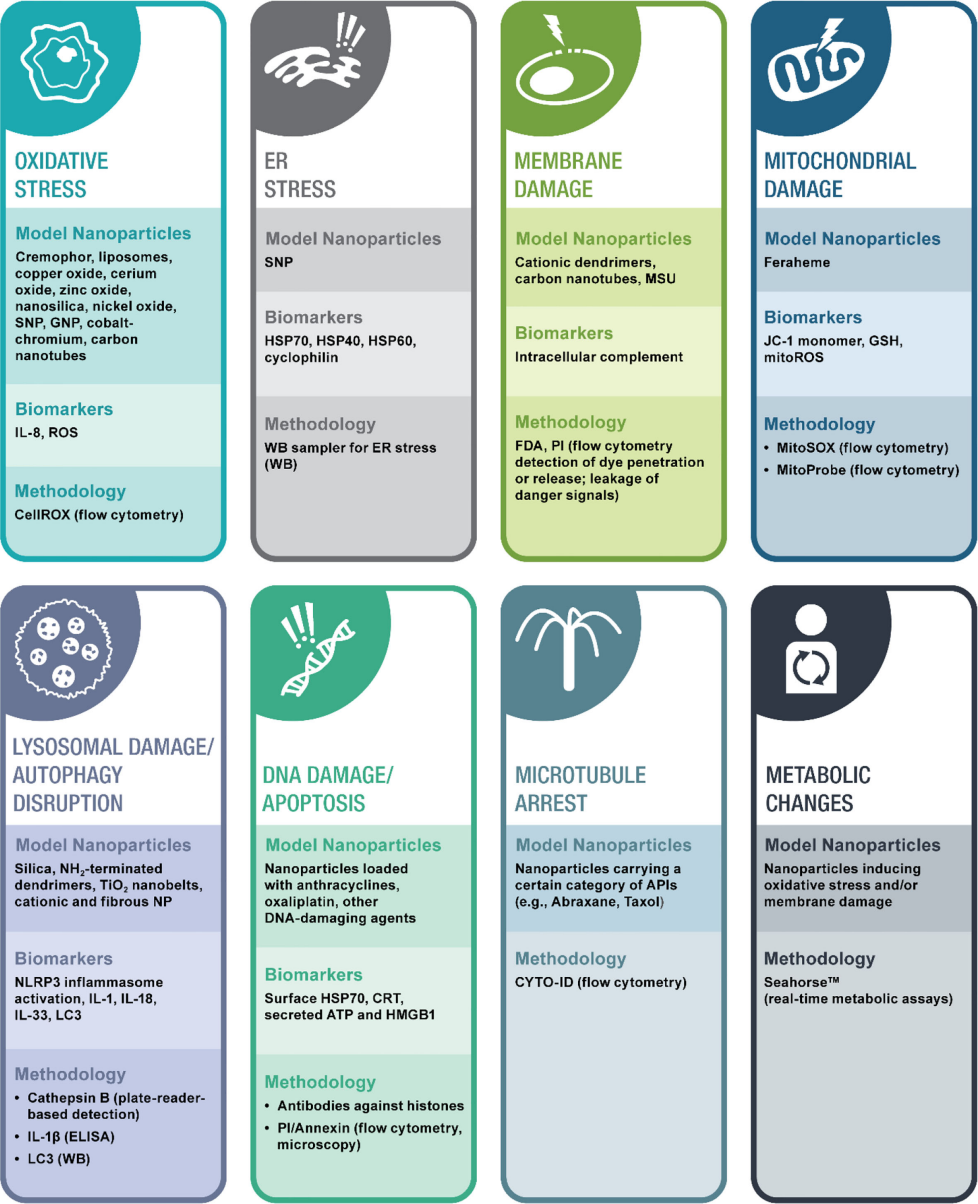
A framework of mechanistic studies. Types of mechanistic studies, model nanoparticles that could be used as controls, methodologies, and intrumentation, and, whenever available, relevant biomarkers are summarized. API, active pharmaceutical ingredient; ATP, adenosine triphosphate; CRT, calreticulin; ER, endoplasmic reticulum; ELISA, enzyme-linked immunosirbent assay; FDA, fluorescein diacetate; GNP, gold nanoparticles; GSH, glutathion; Histones, Phospho-Histone H2AX (Ser139); HSP, heat shock protein; HMG, high mobility group; ID, identifier; LC, light chain; MSU, monosodium urate; NP, nanoparticles; PI, propidium iodine; ROS, reactive oxygen species; SNP, silver nanoparticles; TM, trade mark; WB, western blot.

The investigation of nanoparticle immunological properties progresses toward mechanistic studies involving new technological modalities, such as real-time imaging, advanced immunophenotyping, and immunometabolomics. Some examples of mechanistic studies and relevant methods are summarized in **Figure 9**. The increased use of nucleic acid therapeutics (e.g., mRNA), especially when delivered using nanocarriers with intrinsic pro-inflammatory properties (e.g., LNPs) *via* local routes traditionally used for immunization (e.g., i.m.), in the presence of adjuvants (e.g., TLR agonists, CpG oligos, saponins and other natural products) and intended for use in healthy individuals (e.g., to prevent infections) warrants studies investigating the risk of autoimmunity. Improving *in vitro* and *in vivo* models for assessing nanoparticle immunotoxicity along with harmonization of testing approaches is another important direction in this field. Sharing high quality data generated by “wet” laboratories with bioinformatics researchers is also expected to improve quality of nanotherapeutics, streamline the selection of nanocarriers and aid in developing safer nanomedicines by generating supporting computer-based algorithms and analysis tools.

## Author contributions

The author confirms being the sole contributor of this work and has approved it for publication.

## Funding

This study was funded in whole by federal funds from the National Cancer Institute, National Institutes of Health, under contract 75N91019D00024.

## Acknowledgments

I am grateful to Allen Kane and Karolina Wilk for the help with manuscript preparation.

## Conflict of interest

The author declares that the research was conducted in the absence of any commercial or financial relationships that could be construed as a potential conflict of interest.

## Publisher’s note

All claims expressed in this article are solely those of the authors and do not necessarily represent those of their affiliated organizations, or those of the publisher, the editors and the reviewers. Any product that may be evaluated in this article, or claim that may be made by its manufacturer, is not guaranteed or endorsed by the publisher.

## Publisher’s note

The content of this publication does not necessarily reflect the views or policies of the Department of Health and Human Services, nor does mention of trade names, commercial products, or organizations imply endorsement by the U.S. Government.
